# Innovative Test Strip‐Based Colorimetric Sensors Integrated With Affinity Chromatography: Acetylcholinesterase Inhibitor Screening Breakthrough in *Lycium Barbarum* Leaves

**DOI:** 10.1002/advs.76863

**Published:** 2026-07-29

**Authors:** Yuping Sa, Hui Yuan, Jingui Ma, Zhigang Yang, Mei Wang, Weibiao Wang, Lingling Yang, Fen Ma, Weiman Zhang, Gidion Wilson Mening'oo, Guoning Chen, Xueqin Ma

**Affiliations:** ^1^ School of Pharmacy Ningxia Medical University Yinchuan China; ^2^ Key Laboratory of Protection Development and Utilization of Medicinal Resources in Liupanshan Area College of Pharmacy Ministry of Education Ningxia Medical University Yinchuan China

**Keywords:** acetylcholinesterase, affinity chromatography, alzheimer's disease, colorimetric sensing, lycium barbarum leaves, n‐caffeoylputrescine

## Abstract

Current Alzheimer's drugs exhibit limited effectiveness, highlighting the necessity for multi‐target treatments. This study developed an innovative and efficient screening platform combining hydrogen peroxide test strip‐based colorimetric sensing with affinity chromatography for rapid identification of acetylcholinesterase (AChE) inhibitors from complex herbal medicines. Applying this strategy, from *Lycium barbarum* leaves, we identified three potent inhibitors: chlorogenic acid, N‐acetyl‐N'‐caffeoylputrescine (NANCP), and N‐caffeoylputrescine (NCP), with IC_50_ ranging from 55.7 to 143.2 µm. Molecular analyses confirmed their stable binding to AChE. In a D‐galactose and AlCl_3_‐induced Alzheimer's disease (AD) mouse model, NCP treatment significantly rescued cognitive deficits in AD mice, with the spontaneous alternation rate in the Y‐maze test improved by up to 50%. It markedly reduced cerebral Aβ levels (by 54%) and pro‐inflammatory cytokines, including TNF‐α, IL‐1β, and IL‐6, alleviated oxidative stress, and attenuated hippocampal neuronal damage. Mechanistically, NCP modulated glycerophospholipid metabolism, reshaped gut microbiota, and targeted the proteasome‐autophagy pathway, revealing a multi‐faceted synergistic mechanism. The research offers a new screening tool for AChE inhibitors and highlights a promising natural multi‐target candidate, NCP, for AD therapy.

**Abbreviations**
ACh: Acetylcholine
AChE: Acetylcholinesterase
AD: Alzheimer's Disease
AFM: Atomic Force Microscopy
Aβ: Amyloid‐β
ChOx: Choline Oxidase
DFT: Density Functional Theory
EDS: Energy‐Dispersive Spectroscopy
ELISA: Enzyme‐Linked Immunosorbent Assay
FTIR: Fourier Transform Infrared Spectroscopy
HE: Hematoxylin‐Eosin
HPLC: High‐Performance Liquid Chromatography
HRP: Horseradish Peroxidase
IC_50_
: Half‐Maximal Inhibitory Concentrations
NANCP: N‐acetyl‐N’‐Caffeoylputrescine
NCP: N‐Caffeoylputrescine
OPLS‐DA: Orthogonal Partial Least Squares‐Discriminant Analysis
PBS: Phosphate‐Buffered Saline
PCA: Principal Component Analysis
QC: Quality Control
RMSD: Root Mean Square Deviation
RMSF: Root Mean Square Fluctuation
SEM: Scanning Electron Microscopy
SiO_2_
: Amino‐Silica
TBS: Tris‐Buffered Saline
TCM: Traditional Chinese Medicines
TGA: Thermogravimetric Analysis
TIC: Total Ion Chromatograms
TMB: 3,3',5,5'‐Tetramethylbenzidine
UPLC‐MS: Ultra‐High‐Performance Liquid Chromatography‐Mass Spectrometry
WB: Western Blot
XPS: X‐Ray Photoelectron Spectroscopy

## 1 Introduction

Alzheimer's disease (AD), also referred to as senile dementia, represents the most common type of dementia and is classified as a neurodegenerative disorder [1]. It is characterized by progressive cognitive decline and memory impairment, accompanied by neuronal dysfunction and even neuronal death [2]. Globally, approximately 36.5 million individuals are affected by AD, with China accounting for one‐sixth of the total. As the global population continues to increase, projections indicate that the number of AD patients will surpass 100 million by 2050 [3]. In China, which has the largest population of AD patients, the number is expected to exceed 20 million, with the incidence rate surpassing one‐third among individuals over 85 years of age [4, 5]. As AD poses a significant threat to the health of the elderly, severely impacting their quality of life and imposing heavy medical and social burdens [6], thus requiring urgent attention within the medical field. Although the etiology and mechanism of AD remain unclear [7], numerous studies have identified key pathological features, including the presence of neurofibrillary tangles [8], abnormal tau protein aggregation within neurons [9], and the accumulation of amyloid ‐β (Aβ) in the brain and hippocampus, forming extracellular senile plaques [10]. Among these, Aβ accumulation is of particular concern. Its abnormal aggregation is known to induce mitochondrial dysfunction, oxidative stress, neuroinflammation, and substantial neuronal loss, thereby exacerbating neurodegeneration [11]. Therefore, the rapid and effective clearance of excessive Aβ aggregates in the brains of AD patients is considered a critical therapeutic target [12, 13].

As an intracellular scavenger, autophagy prevents and resolves abnormal protein folding [14], clears damaged and senescent organelles, and phagocytoses dysfunctional intracellular biomacromolecules [15]. Recent studies have shown that defective autophagy in the brains of AD mouse models causes Aβ accumulation, highlighting autophagy's crucial role in Aβ clearance [16]. Reduced levels of the autophagy‐related protein BECN1 correlate with increased cerebral Aβ accumulation in amyloid precursor protein transgenic mice, while elevated BECN1 levels reduce Aβ accumulation [17]. Additionally, Aβ accumulation can induce autophagic dysfunction. Thus, interventions enhancing autophagy may offer an effective therapeutic strategy for combating Aβ and protein aggregation. Due to the complexity of its etiology, none of the current therapeutic regimens can completely cure AD; most merely slow down the progression of the disease [18].

According to the classical cholinergic hypothesis, a fundamental pathogenic factor in AD is reduced cerebral acetylcholine (ACh) concentration, which leads to synaptic transmission dysfunction. Given that ACh is closely linked to learning and memory, increasing cerebral ACh levels is key to improving AD symptoms, with inhibiting AChE activity as the most common strategy [19, 20, 21]. Currently, the majority of clinically approved AD medications are cholinesterase inhibitors (e.g., donepezil) and N‐methyl‐D‐aspartate receptor antagonists (e.g., memantine). However, these drugs can only temporarily alleviate symptoms with limited efficacy and apply solely to patients with mild‐to‐moderate AD, which restricts their widespread clinical use [22, 23, 24, 25]. In recent years, herbal medicines have garnered significant attention due to their potential advantages in the prevention and treatment of AD. Unlike synthetic pharmaceuticals, herbal medicines exert multi‐target, multi‐pathway, and multi‐level integrated regulatory effects, facilitating better symptom improvement [26, 27, 28]. Additionally, herbal medicines are associated with fewer toxic side effects and demonstrate high safety for long‐term use, providing an important direction for the development of novel anti‐AD therapeutics.

In light of these distinctive benefits, this study focuses on the leaves of *Lycium barbarum* L. (LBL), also known as “Tianjing Cao” in the Compendium of Materia Medica, which has been historically documented in Rihuazi Materia Medica for its properties in alleviating vexation, enhancing willpower, and tonifying the body. Contemporary research has identified that LBL contains polysaccharides, polyphenols, alkaloids, etc., with anti‐aging, immune regulation, and sleep‐promoting activities [29, 30]. It is well‐established that the fruits of *L. barbarum* possess anti‐AD effects. Notably, research conducted by Gao Hao's team [31] identified lycibarbarspermidine in *L. barbarum* fruits as key active constituents contributing to its anti‐aging, neuroprotective, anti‐AD, and antioxidant properties. Further studies have suggested that lycibarbarspermidines are characteristic potential markers of medicinal plants in the *Lycium* genus [32, 33]. Importantly, using LC‐MS and other analytical techniques, these lycibarbarspermidine have also been detected in LBL [34], indicating that LBL may exert anti‐AD effects. Ye Minsook et al. [35], proposed that LBL extract could potentially ameliorate memory impairment associated with neurodegeneration. However, to date, limited studies have focused on the anti‐AD pharmacodynamic effects and the corresponding active compounds of LBL, and further investigation is warranted.

**FIGURE 1:**
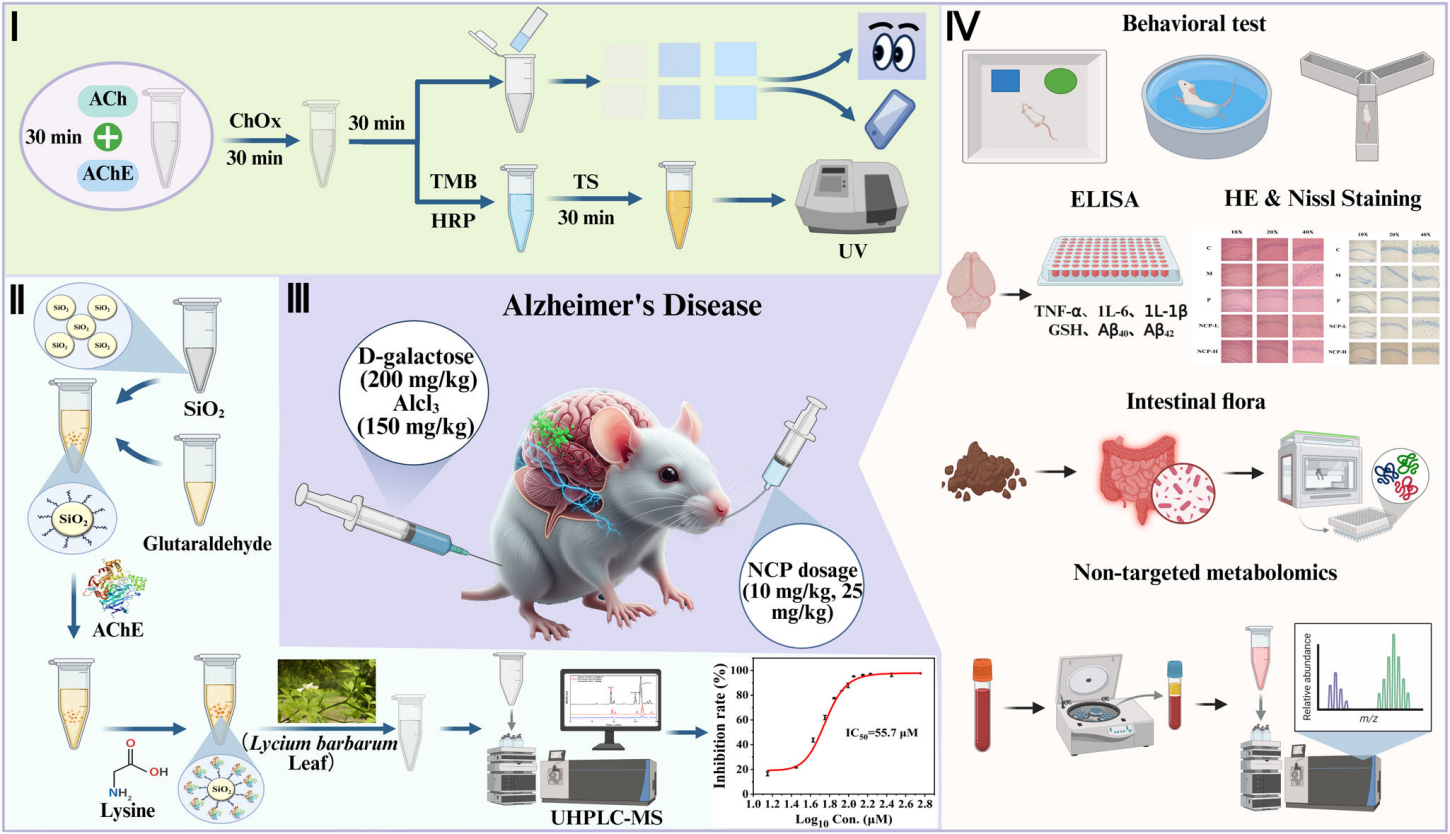
Schematic diagrams illustrating the breakthrough in the screening of AChE inhibitors from LBL, and the research process of the pharmacodynamics of the NCP are presented.

Against this backdrop, developing an efficient, rapid, and low‐cost screening method for AChE inhibitors is critical for novel drug discovery. Traditional methods are hampered by cumbersome operation, low sensitivity, high cost, and poor quantitative accuracy, failing to satisfy drug research and development demands [36, 37, 38]. To resolve this, our team innovatively combined colorimetric sensing with affinity chromatography for screening herbal‐derived AChE inhibitors, a strategy featuring simple operation, cost‐effectiveness, rapid response, environmental friendliness, and visualizable results. First, a rapid screening method was established by combining colorimetric sensors with affinity chromatography technology to obtain potential AChE‐inhibiting active components from LBL. Next, we constructed an AD mouse model induced by D‐galactose and AlCl_3_ to evaluate the in vivo pharmacodynamic effects of the identified inhibitor. Finally, metabolomics and gut microbiota analyses were integrated to elucidate the underlying mechanisms (Figure 1). This study highlights the anti‐AD potential of LBL components, providing new insights and resource support for developing natural‐product‐derived AD therapeutics and investigating their mechanisms.

## 2 Results

### 2.1 Determination of AChE Activity

**FIGURE 2:**
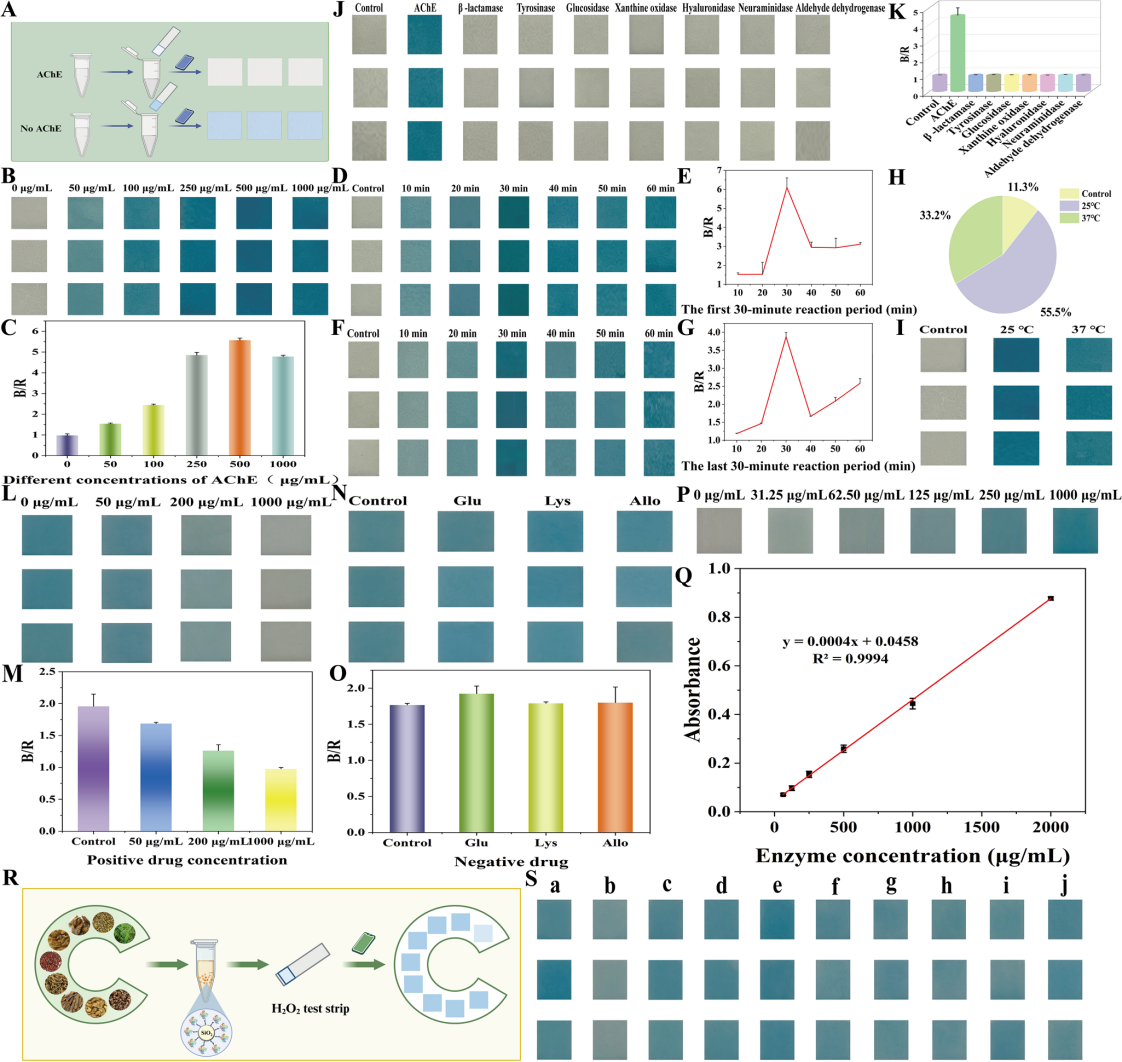
Optimization of different conditions, determination of the standard curve, and screening of herbal medicine inhibitors for AChE colorimetric sensing. (A) Schematic diagram of AChE colorimetric sensing; (B,C) Optimization of different AChE concentrations; (D,E) Optimization of the first 30 min reaction time; (F,G) Optimization of the subsequent 30 min reaction time; (H,I) Optimization of reaction temperature; (J,K) Selectivity analysis of enzymes, including AChE, β‐lactamase, Tyrosinase, Glucosidase, Xanthine oxidase, Hyaluronidase, Neuraminidase, and Aldehyde dehydrogenase; (L,M) Colorimetric sensing results of positive drugs; (N,O) Colorimetric sensing results of negative drugs, from left to right, are: Control, D(+) glucose, L‐lysine, and allopurinol; (P) The intensity range of different colorimetric strips of AChE is 0–1000 µg/mL; (Q) The standard curve and linear equation of AChE; (R) Schematic diagram of herbal medicines inhibitor screening; (S) Colorimetric test strip results of 9 kinds of herbal medicines (a, blank; b, leaves of LBL; c, bitter beans; d, wood fragrance; e, stir‐fried chicken gizzard lining; f, stir‐fried jujube seeds; g, Acacia bark; h, continuity; i, stir‐fried white peony root; j, Qinglong Yi).

The experimental principle is illustrated in Figure 1: AChE catalyzes ACh hydrolysis to produce choline, which, under ChOx action, generates hydrogen peroxide, turning hydrogen peroxide test strips blue. AChE inhibitors suppress hydrogen peroxide production, resulting in no color or light blue strips. Color changes are detectable visually or via smartphone imaging and Photoshop RGB analysis, validating this visual detection method (Figure 2A). This system enables visual AChE detection via enzymatic reaction colorimetric signal changes, providing a technical foundation for subsequent research. Figure 2B,C shows that the colorimetric B/R value increases with AChE concentrations from (0–1000 µg/mL), stabilizing above (500 µg/mL), the optimal concentration. Meanwhile, the B/R value peaks at 30 min post‐reaction (Figure 2D–G), and reaches 55.5% at 25°C (Figure 2H,I). Enzyme selectivity verification revealed significant colorimetric responses only with AChE (Figure 2J,K), confirming good system specificity. To verify biosensor feasibility, clinical donepezil was used as the positive control: B/R values decreased dose‐dependently with increasing donepezil concentrations (Figure 2L,M), indicating the system responds to enzyme activity inhibition. Conversely, negative drug tests showed no significant B/R differences between D‐(+)‐glucose, L‐lysine, allopurinol, and control groups (Figure 2N,O), verifying the system's anti‐interference ability. A colorimetric quantitative method was also established (Figure 1): hydrogen peroxide from AChE‐catalyzed reactions reacts with HRP and TMB; stop solution addition turns TMB from blue to yellow, with absorbance measured at 450 nm. The standard curve (Figure 2P,Q) showed good linearity between absorbance and AChE concentration at 0–2000 µg/mL (y = 0.0004x+0.0458, R^2^ = 0.9994), providing a reliable tool for enzyme activity determination and inhibitor screening. Finally, the optimized system was used to screen 9 herbal medicines for inhibitors. Results showed that LBL extract induced significantly lighter strip color than other groups (Figure 2R,S), indicating potential AChE inhibitory components.

### 2.2 Characterization of AChE@SiO_2_, Screening by Affinity Chromatography Technology and IC_50_ Assay

**FIGURE 3:**
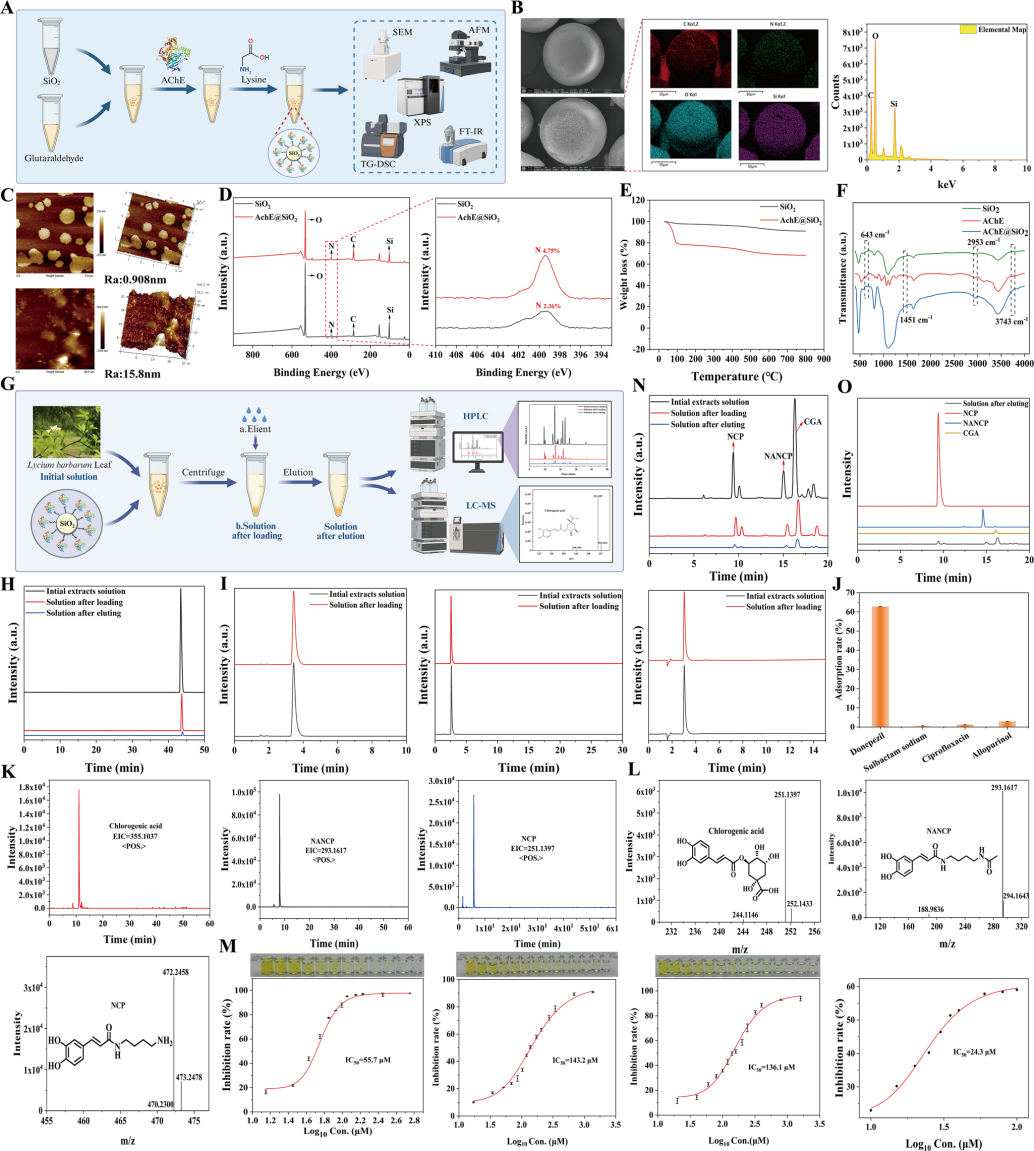
Schematic diagram, characterization, affinity chromatography model screening, and IC_50_ test results of AChE@SiO_2_ synthesis. (A) Schematic diagram of AChE@SiO_2_ synthesis; (B) SEM, EDS and element distribution maps of SiO_2_ and AChE@SiO_2_; (C) 2D and 3D images of the AFM of SiO_2_ and AChE@SiO_2_; (D) XPS spectra of SiO_2_ and AChE@SiO_2_; (E) TGA curves of SiO_2_ and AChE@SiO_2_; (F) FTIR spectra of SiO_2_, AChE and AChE@SiO_2_; (G) Schematic diagram of affinity chromatography model screening of LBL and AChE@SiO_2_; (H) Chromatogram of the interaction between donepezil and AChE@SiO_2_; (I) Chromatograms of the interaction between negative drugs (allopurinol, ciprofloxacin and sodium sulbactam) and AChE@SiO_2_ in sequence from left to right; (J) Histogram of adsorption rates of AChE@SiO_2_ verified with positive drugs and negative drugs. (K) EIC data of these three compounds; (L) MS data of these three compounds; (M) IC_50_ results of three compounds (chlorogenic acid, NANCP, NCP) and donepezil in sequence; (N) Chromatogram of the interaction between LBL and AChE@SiO_2_; (O) HPLC chromatograms of three standard substances and the eluent of LBL.

Figure 3A shows the concept of immobilized enzyme synthesis. The properties of SiO_2_ and AChE@SiO_2_ were characterized to verify AChE immobilization. SEM, energy‐dispersive spectroscopy (EDS), and element distribution maps (Figure 3B) intuitively reveal their morphological differences: SiO_2_ is smooth and spherical, while AChE@SiO_2_ has a rough surface due to enzyme loading; enriched C, O, and Si in AChE@SiO_2_ confirm initial immobilization. AFM images (Figure 3C) show AChE@SiO_2_ has significantly higher surface roughness (Ra = 15.8 nm) than SiO_2_ (Ra = 0.098 nm). XPS analysis (Figure 3D) indicates SiO_2_ contains C, N, O, and Si (N: 2.36%), while AChE@ SiO_2_ has increased N content (4.75%). TGA curves (Figure 3E) show AChE@SiO_2_ has a significant weight loss increase at around 100°C, differing from SiO_2_. Finally, FT‐IR spectrum (Figure 3F) shows AChE@SiO_2_ simultaneously exhibits SiO_2_’s Si‐O characteristic peak (643 cm^−^^1^) and AChE's characteristic peaks (2953 cm^−^^1^, saturated hydrocarbon; 1451 cm^−^^1^, ─CH_2_ bending; 3743 cm^−^^1^, ─NH/─OH stretching). Collectively, SEM, AFM, XPS, TGA, and FT‐IR confirm AChE is successfully immobilized on SiO_2_.

Figure 3G shows the reaction steps between the immobilized enzyme and LBL. Affinity chromatography verified the immobilized AChE screening system's feasibility: HPLC chromatograms (Figure 3H,I) and adsorption rate analysis (Figure 3J) revealed AChE@SiO_2_ adsorbed 63% of the positive drug donepezil, significantly higher than three negative drugs (<3%), confirming the model's reliability. After validation, LBL extract reacted with AChE@SiO_2_; HPLC (Figure 3N) showed successful capture of potential active compounds, which were identified as chlorogenic acid, NANCP, and NCP via UHPLC‐MS (Figure 3K,L) and standard verification (Figure 3O). Activity validation (Figure 3M) indicated concentration‐dependent AChE inhibition for the three monomers (IC_50_: 55.7, 143.2, 136.1 µm) and donepezil (24.3 µm), confirming their good inhibitory effects.

### 2.3 Molecular Docking, Molecular Dynamics Simulation, and DFT

**FIGURE 4:**
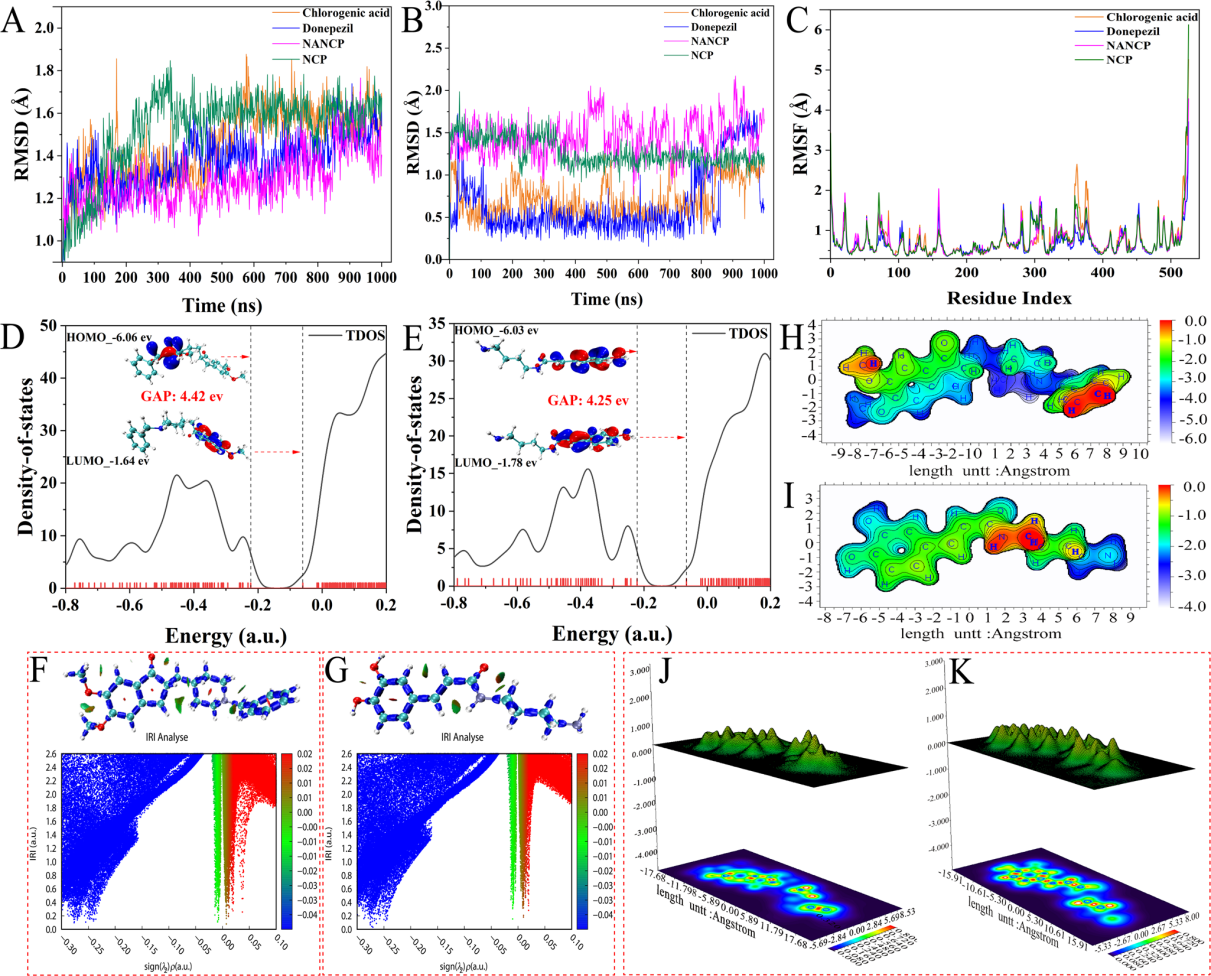
Molecular docking and molecular dynamics simulation study. (A) RMSD spectrograms of proteins; (B) RMSD spectrograms of ligands; (C) RMSF spectrograms of protein; (D,E) respectively depict the total density of states (TDOS) curves of donepezil and NCP, along with the electron cloud distributions and corresponding energy levels of their HOMO and LUMO orbitals; (F,G) show the IRI analysis plots of donepezil and NCP, respectively. Based on the distributions of sign(λ_2_)ρ and IRI values, these plots characterize the regional features of different weak interactions within the systems (blue regions in the plots correspond to sign(λ_2_)*ρ* < 0, representing strong attractive interactions such as hydrogen bonds; green regions correspond to sign(λ_2_)*ρ* ≈ 0, representing weak van der Waals interactions; red regions correspond to sign(λ_2_)*ρ* > 0, representing repulsive interactions); (H, I) present the surface distance projection maps of donepezil and NCP, respectively; (J, K) The electron density map and its 3D projection of donepezil and NCP.

To further explore the binding mechanism and interaction stability between active monomers and AChE, molecular docking was performed to predict the binding modes, and dynamic modeling was used to verify the dynamic conformational characteristics of the complexes. As shown in Figure S1A–D, donepezil binds AChE via interactions with TYR‐337, PHE‐295, LEU‐289, VAL‐294, and TRP‐86; chlorogenic acid interacts with TYR‐124 and TRP‐286; NANCP forms specific interactions with ALA‐204 and ILE‐451; NCP binds through interactions with TYR‐341, PHE‐297, and VAL‐294. These interactions provide a preliminary structural basis for their inhibitory activity. To evaluate the dynamic stability of the complexes, 1000 ns MD simulations were carried out. Root mean square deviation (RMSD) results (Figure 4A,B) showed that RMSD fluctuations of all protein‐ligand complexes (donepezil and three active components with AChE) were <1 Å during the simulation; protein and ligand RMSD values were stably maintained at 1–1.6 Å and 0.5–1.5 Å, respectively, indicating stable conformational complexes. Subsequently, root mean square fluctuations (RMSF values were calculated to assess AChE residue fluctuation. Figure 4C shows consistent RMSF curve trends for all ligands, with residue fluctuation amplitudes stably <6 Å, confirming low residue flexibility and overall complex stability. Protein‐ligand interaction analysis (Figure S1E–H) revealed that donepezil binds via hydrogen bonds with AChE's PHE‐295; chlorogenic acid forms continuous, stable, strong hydrogen bonds with GLY‐122, TYR‐124, and HIS‐447; NANCP with GLY‐121, GLY‐122, TYR‐124, SER‐203, and HIS‐447; NCP via hydrogen bonds with GLU‐202. In summary, molecular docking and MD simulations confirm that donepezil and the three active components efficiently bind AChE with good dynamic stability, providing molecular‐level support for their AChE inhibitory effects and verifying the screening model's reliability.

Subsequently, DFT calculations were performed to systematically investigate the electronic structural information and weak interaction characteristics of donepezil and NCP. As illustrated in Figure 4D,E, the HOMO‐LUMO energy gap of donepezil was 4.42 versus 4.25 eV for NCP (with NCP showing a narrower gap and a lower electron transition barrier), and the two compounds exhibited significant differences in the localization of frontier orbitals. IRI analysis (Figure 4F,G) revealed that donepezil had more concentrated intramolecular hydrogen bonding interactions, whereas NCP was dominated by weak van der Waals interactions, thus directly distinguishing the types and proportions of weak interactions between the two molecules. As shown in Figure 4H,I, the isosurface of donepezil displayed localized “bulging” red high‐value regions, reflecting its high local steric hindrance and atomic crowding, while NCP featured low steric hindrance and an extended structure. The localized orbital locator (LOL) plots (Figure 4J,K) further verified the regional specificity of electron density. Collectively, these results elucidate the microscopic differences between the two compounds from the perspectives of electronic structure and weak interactions, providing a theoretical basis for interpreting the correlation between their physicochemical properties and biological activities.

### 2.4 In Vivo Anti‐AD Pharmacodynamic Effects

**FIGURE 5:**
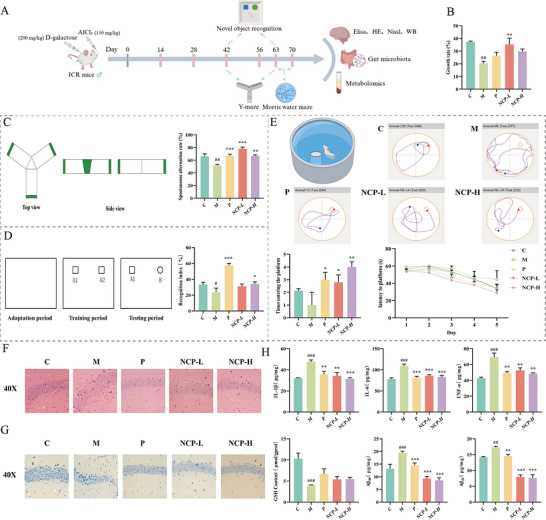
Pharmacodynamic evaluation of the alkaloid component NCP from LBL against AD in vivo. (A) Schematic diagram of zoological experiment; (B) Effect of NCP administration on body weight of mice in each group (n = 8); (C) Y‐maze test to explore the effect of NCP on spontaneous alternation rate of AD mice (n = 8); (D) Novel object recognition test to study the effect of NCP on novel object recognition ability of AD mice (n = 8); (E) Morris water maze test to investigate the effect of NCP on escape latency and number of platform crossings of AD mice (n = 8); (F) Effect of NCP on nerve cells in the CA1 region of the hippocampus of mice in each group (n = 8); (G) Effect of NCP on the number of Nissl‐positive cells in the CA1 region of the hippocampus of mice in each group (n = 8); (H) Effect of NCP on the contents of inflammatory factors (TNF‐α, IL‐1β and IL‐6), oxidative stress factor (GSH), Aβ_40_ and Aβ_42_ in brain tissue of mice in each group (n = 8). (^*^ *p* <0.05, ^**^ *p* <0.01, ^***^ *p* <0.001, vs. M; ^#^ *p* <0.05, ^##^ *p* <0.01, ^###^ *p* <0.001, vs. C). All datasets passed the normality test, and thus, parametric tests were adopted for statistical analysis in this study. Data are shown as mean ± SD. For multiple comparisons, one‐ way ANOVA followed by Dunnett's post hoc test was performed on Graphpad Prism software. All experiments were repeated at least three times.

Given that the AChE inhibitor donepezil is clinically utilized for the treatment of AD, the AChE inhibitor compounds identified in our study may also exhibit anti‐AD effects. As shown in Figure 3N and the quantitative results, chlorogenic acid had the highest content in the LBL extract, followed by NCP, while NANCP was present at a relatively low level. The low yield of NANCP made it impossible to support large‐scale animal experiments. In terms of AChE inhibitory activity, the IC_50_ values of chlorogenic acid, NANCP, and NCP were 55.7, 143.2, and 136.1 µm, respectively. Although chlorogenic acid exhibited the strongest inhibitory effect, its anti‐AD pharmacological activities have been extensively reported in previous studies [39]. In comparison, NCP is a characteristic component of LBL with potent AChE inhibitory activity, which is superior to that of NANCP. Taken together, NCP possesses moderate inhibitory activity and relatively high natural abundance, making it the optimal candidate for further investigation in this work. A schematic diagram of the animal pharmacodynamic experiment is shown in Figure 5A. After 10 weeks of administration, the model group had significantly lower body weight than the control (*p* <0.01), and the NCP‐L group had a significant weight increase (*p* <0.01, Figure 5B). These results indicate NCP improves model‐induced weight loss with good safety.

On the basis of this safety verification, three behavioral tests were conducted to evaluate the improving effect of NCP on cognitive dysfunction in AD mice. Y‐maze test (Figure 5C) showed the model group had a significantly lower spontaneous alternation rate than the control (*p* <0.01), confirming successful AD mouse modeling; the NCP groups and positive drug group significantly increased this index (*p* <0.001 or *p* <0.01), reversing working memory deficit. Novel object recognition test (Figure 5D) revealed the model group had reduced discrimination ability compared to the control (*p* <0.05); the positive drug group and NCP‐H group improved cognitive ability (*p* <0.001 or *p* <0.05), indicating that NCP could restore the novel object discrimination ability. As the gold standard for spatial learning and memory evaluation, the Morris water maze test (Figure 5E) complemented the above results: compared to the control, the model group had fewer platform crossings, longer escape latency and complex trajectories; NCP groups and positive drug group significantly increased platform crossings (*p* <0.01 or *p* <0.05), with trajectories preferring the target quadrant and shortened escape latency. In summary, NCP significantly protects AD mice against working memory, novel object discrimination, and spatial memory impairments.

The CA1 region of the hippocampus is a pathologically sensitive brain region in AD, and the integrity of its neurons is associated with cognitive function. To clarify the neuroprotective effect of NCP, hematoxylin and eosin (HE) and Nissl staining were performed. HE staining (Figure 5F) showed that compared with the control group, the model group had reduced, disordered hippocampal CA1 neurons with nuclear necrosis; NCP intervention increased neuron number, improved arrangement regularity, and alleviated necrosis. Nissl staining (Figure 5G) revealed that the model group had significantly reduced, disordered Nissl body‐positive cells compared to the control group; the NCP groups and positive drug group showed increased Nissl bodies and neat cell arrangement. In summary, NCP improves hippocampal CA1 neuronal pathological damage and maintains Nissl body stability in AD mice, exerting neuroprotective effects and providing histopathological evidence for its cognitive protection.

Neuroinflammation, oxidative stress disorders, and Aβ deposition are the core pathological processes of AD. To clarify NCP's neuroprotective mechanism, inflammatory factors (TNF‐α, IL‐1β, IL‐6), oxidative stress factor (GSH), Aβ_40_, and Aβ_42_ in mouse brain tissues were detected (Figure 5H). Compared with the control group, the model group had significantly increased inflammatory factors and Aβ (*p* <0.001) and decreased GSH (*p* <0.001). NCP inhibited inflammatory factors and Aβ accumulation (*p* <0.001 or *p* <0.01) and tended to increase GSH, suggesting it acts by improving these interrelated core AD pathological cascades. In summary, NCP has significant in vivo anti‐AD activity, ensuring safety, reversing multi‐domain cognitive deficits, protecting hippocampal neurons, and targeting core AD pathology, providing important experimental evidence for its potential as an anti‐AD candidate drug.

### 2.5 Untargeted Metabolomics

**FIGURE 6:**
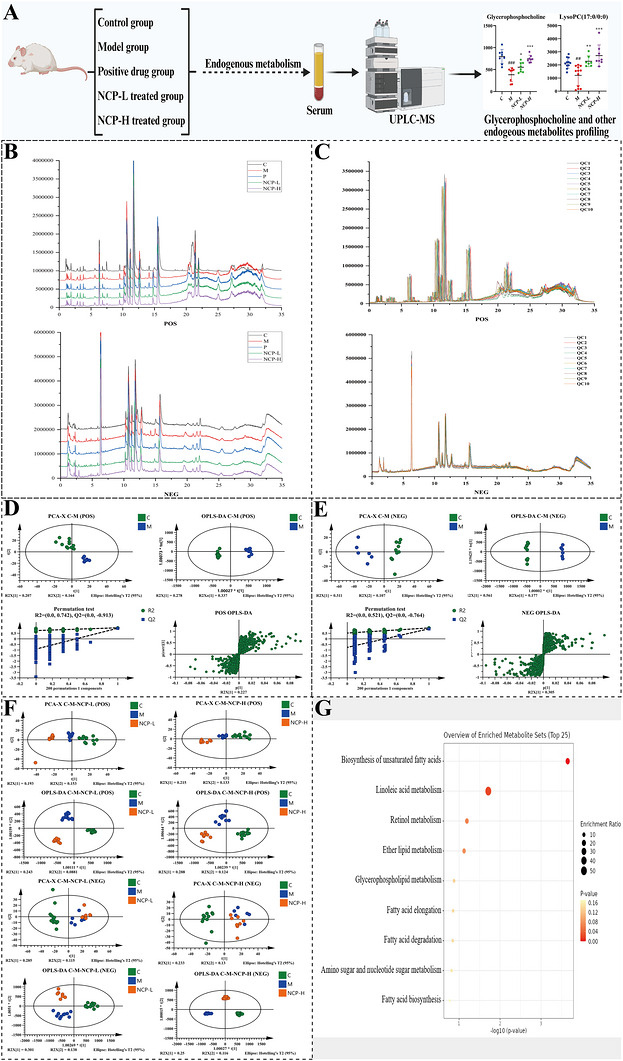
Untargeted metabolomics study of the alkaloid component NCP from LBL. (A) Schematic diagram of untargeted metabolomics experiment; (B) TIC of mouse serum in positive and negative ion modes; (C) TIC of all QC samples in positive and negative ion modes; (D,E) PCA, OPLS‐DA, permutation test and OPLS‐DA score plots of the control group and the model group in positive and negative ion modes; (F) PCA and OPLS‐DA plots after administration in positive and negative ion modes; (G) Bubble plot of differential metabolite pathway analysis.

To systematically clarify the regulatory effect and mechanism of NCP on AD mice's endogenous metabolism, untargeted metabolomics analysis was performed (Figure 6A). UPLC‐MS detection of each group's mouse serum yielded clear total ion chromatograms (TIC) in positive and negative ion modes (Figure 6B), and the chromatograms of quality control (QC) samples showed good overlap (Figure 6C), confirming detection stability and reliability. Multivariate statistical analysis showed significant separation between control and model groups in principal component analysis (PCA) under both ion modes (Figure 6D,E); orthogonal partial least squares‐discriminant analysis (OPLS‐DA) models (positive: R^2^ = 0.742, Q^2^ = −0.913; negative: R^2^ = 0.521, Q^2^ = −0.764) and permutation tests verified intergroup metabolic profile differences. After NCP intervention, the administration group serum metabolites tended to revert to the control group (Figure 6F), indicating NCP reversed abnormal metabolic disorders. Via OPLS‐DA (VIP > 1) and independent samples t‐test (*p* <0.05), 392 AD‐related differential ions were screened (198 positive, 194 negative). Secondary mass spectrometric identification, database comparison, and literature analysis ultimately identified 56 differential metabolites (39 and 17 in positive and negative ion mode, respectively), including lysophosphatidylcholine, glycerophosphocholine, and fatty acids. Detailed information is provided in Tables S1 and S2.

The 56 differential metabolites were imported into MetaboAnalyst for pathway analysis (Figure 6G). The results showed that 9 pathways were abnormal in AD mice, including unsaturated fatty acid biosynthesis and linoleic acid metabolism. Among them, unsaturated fatty acid biosynthesis, linoleic acid metabolism, and glycerophospholipid metabolism were the main abnormal pathways. Further analysis (Figures S2, S3) revealed that NCP could significantly regulate 25 differential metabolites, especially restoring the levels of key metabolites such as lysophosphatidylcholine and glycerophosphocholine in the glycerophospholipid metabolism pathway, suggesting that NCP may improve AD metabolic disorders by regulating this pathway. It is known that abnormalities in the glycerophospholipid metabolism pathway are closely related to autophagy regulation, and the imbalance of its metabolic homeostasis may participate in the pathological process of AD by affecting autophagic flux [40]. This provides an important direction for subsequent exploration of the anti‐AD effect of NCP by regulating glycerophospholipid metabolism‐mediated autophagic pathway.

### 2.6 Intestinal Flora

**FIGURE 7:**
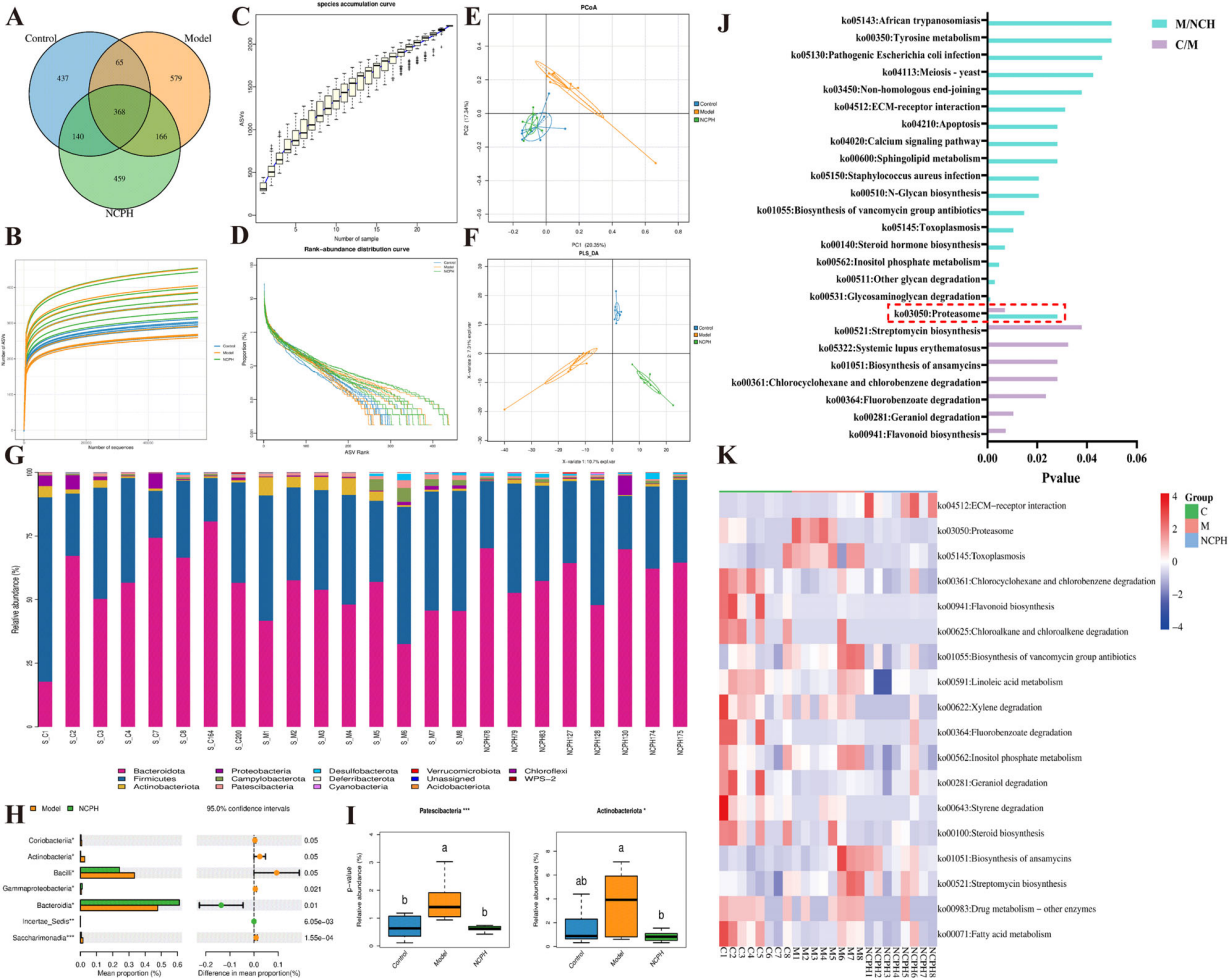
Study on the Effect of NCP on the Intestinal Flora of AD Mice. (A) Comparison of OTU/ASV distribution with Venn diagram; (B) Species accumulation curve; (C) Rank‐abundance curve; (D) Shannon‐Wiener curve; (E) Principal coordinate analysis (PCoA) for calculating the β diversity of the three groups of mice; (F) Partial least square discriminant analysis (PLS‐DA) for calculating the β diversity of the three groups of mice; (G) Bar chart of community structure analysis at the phylum level; (H) Analysis of the abundance of different intestinal flora categories and inter‐group differences in the two groups of samples; (I) Box plot of community structure analysis at the phylum level; (J) Prediction of intestinal flora metabolic function by PICRUSt in the blank group, model group and administration group; (K) Heatmap of the relative abundance of intestinal flora functional pathways in samples from different groups.

To clarify NCP's regulatory effect on the intestinal flora of AD model mice, multi‐dimensional flora analysis was performed to reveal structural and functional remodeling characteristics. OTU/ASV Venn distribution (Figure 7A) showed significant intergroup flora differences: the AD model group shared only 65 OTU/ASV with the control (extremely low overlap) and had more unique OTU/ASV (579 vs. 437 of the control), indicating severe AD‐induced intestinal flora disruption. Compared with the model group, the NCPH group had fewer unique OTU/ASV (459) and more shared with the control (140), suggesting NCP restored core flora and normal flora structure overlap. Rarefaction curves (Figure 7B) tended to saturate with increased sequencing depth, and species accumulation curves (Figure 7C) further quantified this trend. Rank‐abundance curves (Figure 7D) showed the model group had a steeper declining curve with reduced flora abundance/evenness, while the NCPH group had a gentler slope and long tail, indicating improved diversity. *β*‐diversity analysis (PCoA and PLS‐DA, Figure 7E,F) showed clear clustering and separation of the three groups, with NCPH group samples approaching the control, further confirming NCP could reverse the disrupted intestinal flora structure of AD mice.

Furthermore, the flora structure was analyzed at six taxonomic levels (phylum, class, order, family, genus, species). At the phylum level, fluctuations in core taxa (e.g., Bacteroidota, Firmicutes) were associated with the AD phenotype (consistent with the characteristic of “disrupted Bacteroidetes–Firmicutes ratio”). Actinobacteria abundance was significantly increased in the AD model group, a trend reversed by the NCPH group (Figure 7G,I). At the class level, abundance differences in dominant classes (e.g., Bacteroidia, Bacilli) reflected flora functional imbalance (Figure 7H). At the order level, the model group showed decreased Bacteroidales and Lactobacillales abundances, along with increased Bacillales, Erysipelotrichales, and Clostridiales, indicating a shift in niche competition between symbiotic bacteria and opportunistic pathogens. At the family level, the model group exhibited decreased Muribaculaceae, Lactobacillaceae, and Bacteroidaceae; increased Bacillaceae, Clostridiaceae, and Erysipelotrichaceae; changes significantly reversed by NCP. These disorders were verified at the genus and species levels: the model group had decreased Muribaculaceae‐genus, Lactobacillus, and Bacteroides; abnormal enrichment of Bacillus, Bifidobacterium, and Lactobacillus_vaginalis, forming an AD‐related multi‐taxonomic synergistic disorder network of intestinal flora. Such disorders may exacerbate neurodegeneration by transmitting metabolite signals (e.g., endotoxins, short‐chain fatty acids) via the “gut‐brain axis,” and provide multi‐level potential targets for gut flora‐targeted AD intervention (Figures S4–S15).

After 16S rDNA sequencing, PICRUSt analysis was performed to predict the flora function and explore NCPH's intervention effect on AD. Differential metabolic pathways were compared between control vs. model and model vs. NCPH groups (*p*<0.05, Figure 7J): 8 significant differential pathways existed between control and model (e.g., proteasome, streptomycin biosynthesis enrichment); 18 between model and NCPH (e.g., African trypanosomiasis, tyrosine metabolism). The two pairwise comparisons are co‐enriched in the proteasome pathway (involved in the ubiquitin‐proteasome pathway, UPP). UPP and the autophagic pathway are core cellular protein degradation systems with close interactive regulation; UPP dysfunction may participate in AD pathology via abnormally folded protein accumulation, laying a theoretical foundation for subsequent exploration of NCP's anti‐AD effect through regulating gut flora‐mediated UPP‐autophagy interactive network [41, 42]. Additionally, clustering differences between NCPH and model groups in the functional pathway relative abundance heatmap (Figure 7K) verified this trend.

### 2.7 Western Blot

**FIGURE 8:**
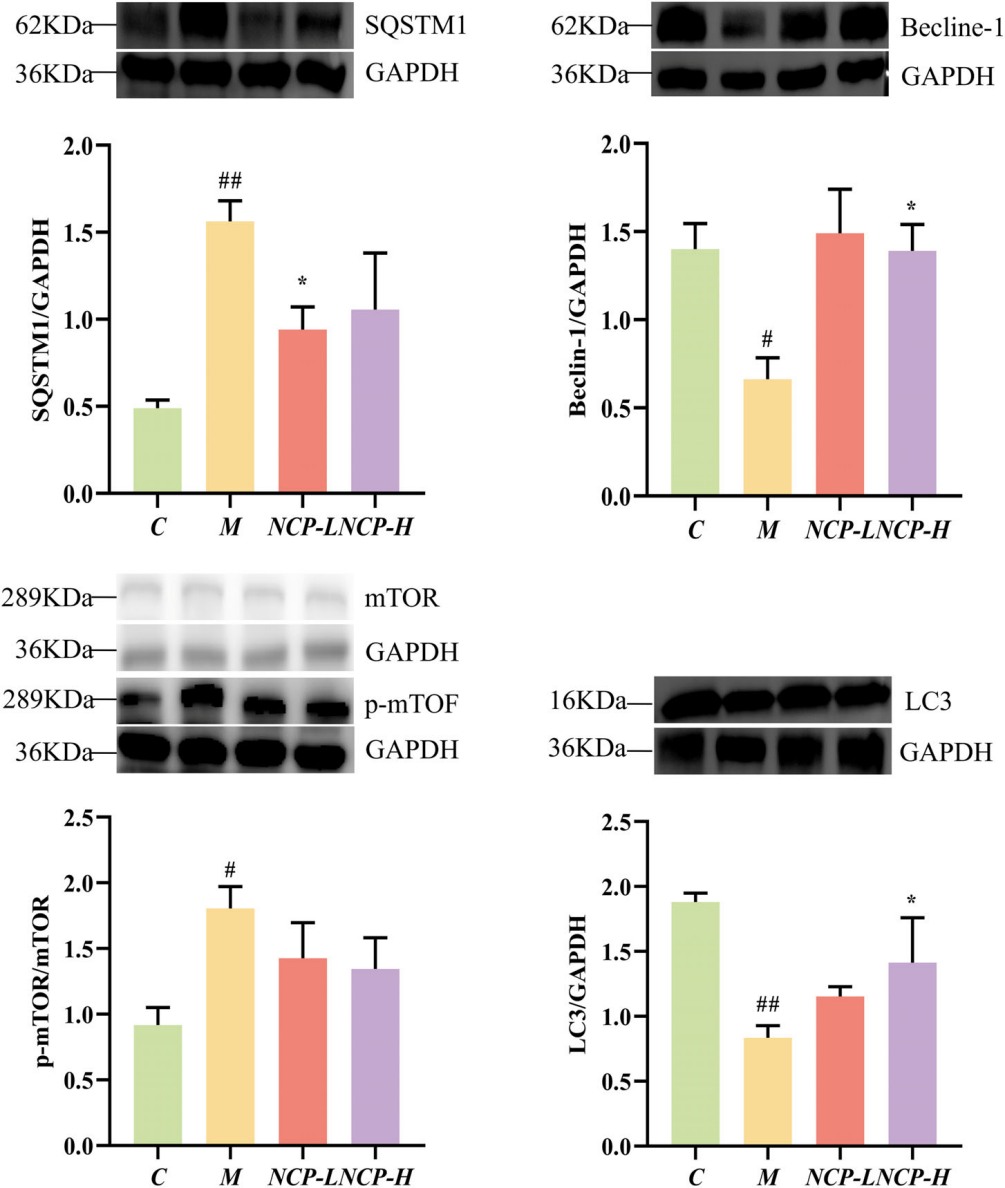
Effect of NCP on contents of SQSTM1, Beclin‐1, LC3, and p‐mTOR/mTOR in brain tissue of AD mice (n = 3). (^*^ *p* <0.05, ^**^ *p* <0.01, ^***^ *p* <0.001, vs. M; ^#^ *p* < 0.05, ^##^ *p* < 0.01, ^###^ *p* < 0.001, vs. C). All datasets passed the normality test, and thus, parametric tests were adopted for statistical analysis in this study. Data are shown as mean ± SD. For multiple comparisons, one‐ way ANOVA followed by Dunnett's post hoc test was performed on Graphpad Prism software. All experiments were repeated at least three times.

To verify metabolomics and gut microbiota results, clarify the correlation between autophagy‐related proteins and AD‐induced brain damage, and combine pathway inference, Western blot detected SQSTM1, Beclin‐1, LC3, mTOR, and p‐mTOR in mouse brain tissues (Figure 8). Compared with the control group, the model group had significantly increased SQSTM1 and p‐mTOR/mTOR (*p*<0.01 or *p*<0.05) and decreased Beclin‐1 and LC3 (*p*<0.01 or *p*<0.05), suggesting these proteins may mediate AD brain damage. Compared with the model group, the NCP‐L group showed significantly reduced SQSTM1 (*p*<0.05), a downward trend in p‐mTOR/mTOR, and significantly increased Beclin‐1 and LC3 (*p*<0.05), indicating NCP exerts neuroprotective effects by regulating these autophagy‐related proteins.

## 3 Discussion

**FIGURE 9:**
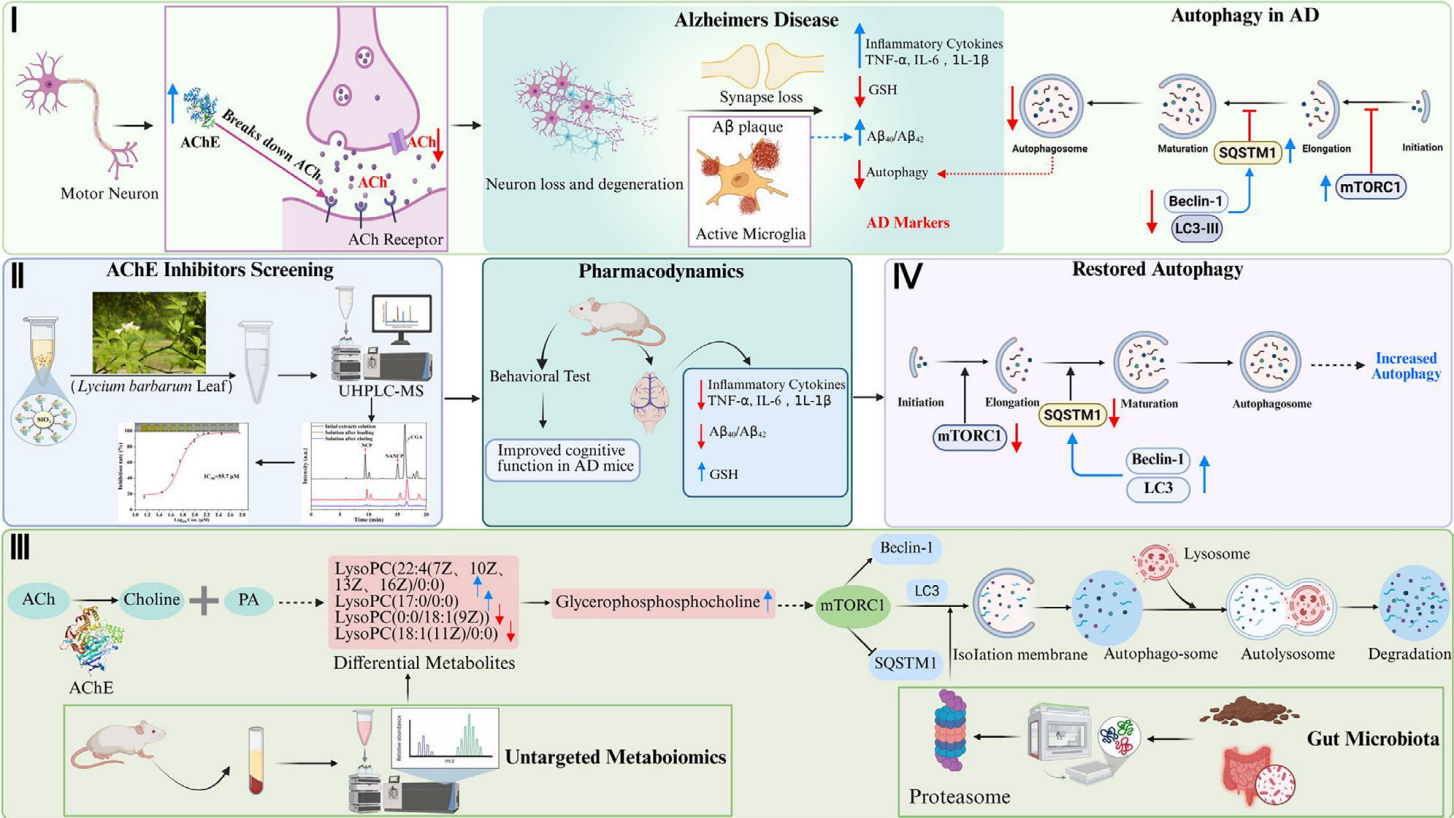
This figure illustrates the mechanistic study from the screening of AChE inhibitors in LBL to their improvement of cognition in AD mice, as well as metabolomics analysis associated with intestinal flora.

AD is an insidious‐onset, progressive neurodegenerative disease. Early patients have mild memory decline; as the disease progresses, they develop severe cognitive impairments, eventually losing self‐care ability and burdening families and society [43, 44]. Current clinical drugs only relieve symptoms with obvious adverse reactions; herbal medicine, including traditional Chinese medicines (TCM), has low toxicity but unclear anti‐AD pharmacodynamic substances and mechanisms [45]. Based on this, this study screened active components from LBL and analyzed their anti‐AD mechanisms, with the core action mode intuitively presented in the final mechanism diagram (Figure 9).

We employed a D‐galactose/AlCl_3_‐induced mouse model of Alzheimer's disease (AD) instead of the common APP/PS1 and 5xFAD transgenic models, which are costly and require extensive maintenance. The D‐galactose/AlCl_3_ model, despite lacking the genetic mutations and severe pathology of familial AD, is a validated and widely used model for studying sporadic, age‐related cognitive decline [46, 47]. It effectively replicates key pathological features of sporadic AD, such as cholinergic system disorder, redox imbalance, neuroinflammation, Aβ buildup, and progressive cognitive impairment [48, 49]. This model is advantageous for screening bioactive compounds from natural products due to its short duration, low cost, stable pathology, and high reproducibility, making it ideal for testing herbal medicine ingredients.

A core pathology of AD is cholinergic neurotransmission dysfunction: AChE‐mediated excessive hydrolysis of synaptic cleft ACh reduces its level, triggering cognitive impairment and accelerating disease progression [50]. Inhibiting AChE is a key anti‐AD direction, but traditional methods cannot rapidly screen active components from herbal medicines. Thus, this study constructed a novel screening technology that realizes visual and quantitative AChE activity analysis via hydrogen peroxide test strip color changes. Concentration‐dependent color fading of the positive drug donepezil and stable negative drug performance verified the method's specificity and reliability. Herbal medicines screening showed LBL extract had AChE inhibitory activity. Further, AChE@SiO_2_ affinity chromatography‐specific adsorption combined with LC‐MS identified three components (chlorogenic acid, NANCP, and NCP). IC_50_ determination (55.7∼143.2 µm) confirmed their good inhibitory ability, indicating LBL contains potential AChE inhibitors. This study further explores the active substance basis of three AChE inhibitors isolated from *Lycium barbarum* leaves. Chlorogenic acid, NANCP, and NCP all contain a caffeoyl moiety, which acts as the core functional group for AChE inhibition. The conjugated π‐system and phenolic hydroxyl groups within the caffeoyl structure can form hydrogen bonds and hydrophobic interactions with amino acid residues in the active pocket of AChE, which is the key factor contributing to their inhibitory effects. Among the three compounds, chlorogenic acid exhibits the strongest AChE inhibitory activity with an IC_50_ value of 55.7 µm. This may be attributed to its multiple phenolic hydroxyl groups and quinic acid skeleton, which could provide additional binding sites and strengthen interactions with the enzyme. Both NCP and NANCP are aliphatic amine derivatives with a putrescine skeleton. The long putrescine chain can insert into the hydrophobic cavity of AChE, while the terminal caffeoyl group serves as the major binding site. A slight difference in inhibitory potency was observed between NCP (136.1 µm) and NANCP (143.2 µm). It is speculated that the N‐acetyl group on NANCP may increase steric hindrance at the binding region, weaken its binding affinity for AChE, and consequently lead to a slight decline in inhibitory activity. Combined with molecular docking results, the above structural features are consistent with the binding modes of the three compounds to AChE. This study preliminarily reveals the structure‐activity relationship of caffeoyl derivatives against AChE, and helps to elucidate the potential active substance basis responsible for the anti‐Alzheimer's activity of *Lycium barbarum* leaves. Finally, compared with traditional methods, this colorimetric sensing‐affinity chromatography combined technology has the advantages of visualization, rapidity, high efficiency, low cost, and high specificity. It provides a new tool for AChE inhibitor screening in complex herbal medicine systems and is expected to accelerate AD therapeutic drug development.

Existing studies have shown that LBL extract [51] and chlorogenic acid [52] have neuroprotective potential, and LBL extract can improve cognition in AD mice, but none have clarified the pharmacodynamic substances and mechanisms. Since chlorogenic acid's anti‐AD activity has been confirmed in the literature, and the laboratory‐synthesized NANCP has a limited yield that cannot meet the in vivo pharmacodynamic experiment needs, this study conducted systematic pharmacodynamic verification on NCP. Y‐maze, novel object recognition, and Morris water maze tests confirmed that NCP improved working memory and spatial learning ability in AD mice, indicating neuroprotective effects. Mechanism studies showed that NCP down‐regulated cerebral inflammatory factors and Aβ_40_ and Aβ_42_, and tended to increase oxidative stress indicators, suggesting its anti‐AD effect is related to inhibiting neuroinflammation, reducing Aβ deposition, and regulating oxidative balance. Pathological staining further revealed that NCP improved neuronal atrophy, reduced nuclear pyknosis, restored Nissl body number and distribution, and repaired neuropathological damage. In summary, multi‐dimensional experiments confirmed NCP's anti‐AD efficacy, providing an experimental basis for its in‐depth development and mechanism analysis.

Second, in AD pathogenesis, metabolic pathway disorders are closely associated with neuronal dysfunction and cognitive impairment. Untargeted metabolomics identified 56 highly sensitive and specific differential metabolites, which were mainly enriched in 9 AD‐related pathways (e.g., unsaturated fatty acid biosynthesis, linoleic acid metabolism, glycerophospholipid metabolism). Among them, 25 differential metabolites could be significantly regulated, especially 6 key ones in the glycerophospholipid metabolism pathway (e.g., lysophosphatidylcholine (LysoPC), glycerophosphocholine). Of these key metabolites, 4 (glycerophosphocholine, LysoPC(17:0/0:0), LysoPC(22:4(7Z,10Z,13Z,16Z)/0:0), LysoPC(P‐16:0/0:0)) were upregulated in AD, and 2 (LysoPC(18:1(11Z)/0:0), LysoPC(0:0/18:1(9Z))) were downregulated, further confirming the association between AD and glycerophospholipid metabolism disorders. Numerous existing studies have confirmed that glycerophospholipid metabolism imbalance is a core metabolic characteristic of AD. For example, Hannah R. Bulgart et al. [53], found significantly decreased LysoPC(18:1) levels in AD patients’ cerebrospinal fluid, which was positively correlated with hippocampal neuron membrane integrity disruption and cognitive scores. In addition, significant upregulation of glycerophosphocholine in AD patients’ cerebral cortex was suggested to be closely related to neuronal membrane structural integrity loss [54], consistent with the metabolite trend of AD model mice in this study. Meanwhile, Previous studies have shown [55, 56] that abnormal elevation of polyunsaturated fatty acid‐type lysophosphatidylcholines in APP/PS1 transgenic AD mice exacerbates Aβ deposition by activating microglial inflammation, which explains the decreased inflammatory factor levels after NCP regulates these metabolites in this study. Notably, the interaction between glycerophospholipid metabolism and autophagy has become an AD mechanism research hotspot. Existing studies [57, 58, 59] indicated that glycerophospholipids are core autophagosomal membrane components, and their metabolite glycerophosphocholine affects autophagy initiation via regulating the mTOR pathway; its abnormal accumulation in AD activates mTORC1 phosphorylation, inhibiting autophagic flux initiation. In contrast, dysregulated LysoPC disrupts autophagosomal membrane fluidity and hinders autophagosome‐lysosome fusion [60]. This echoes the study's results: after NCP regulates key glycerophospholipid metabolites, p‐mTOR/mTOR and SQSTM1 levels decrease, while autophagy‐related proteins Beclin‐1 and LC3 are upregulated in AD mice. These suggest NCP restores glycerophospholipid metabolic homeostasis, relieves autophagic pathway inhibition, and ultimately promotes normal autophagic flux to clear Aβ deposition.

In recent years, the mechanism by which gut microbiota participates in AD pathogenesis via the gut‐brain axis has attracted wide attention. Its imbalance can induce intestinal barrier damage, chronic inflammation, and metabolic disorders, thereby affecting neurotransmitter synthesis and brain homeostasis. 16S rDNA sequencing showed that the gut microbiota structure of AD model mice was imbalanced (low overlap with the control group, more unique units, decreased diversity) and exhibited “beneficial bacteria reduction and harmful bacteria enrichment”; NCP improved these abnormalities and promoted the microbiota to approach a normal state. At multiple taxonomic levels, beneficial microbiota abundance was generally downregulated: Muribaculaceae, Lactobacillaceae, Bacteroidaceae, and their corresponding orders, genera, and species (beneficial intestinal symbionts) maintain intestinal barrier integrity and inhibit inflammation by producing short‐chain fatty acids, and their decreased abundance has been repeatedly confirmed in AD patients and animal models (e.g., significantly reduced Lactobacillus in AD patients’ intestines [61]. In contrast, opportunistic pathogens (Bacillaceae, Clostridiaceae, Erysipelotrichaceae, and their corresponding taxa) were significantly enriched; their excessive proliferation damages the intestinal barrier, releases endotoxins, induces neuroinflammation via the gut‐brain axis, and is closely related to AD pathological progression [62]. At the phylum level, model mice showed a disrupted Bacteroidota/Firmicutes ratio and increased Actinobacteriota abundance. This is consistent with classic AD gut microbiota conclusions [63], further confirming the reliability of model construction and rationality of this study's results. Microbiota function prediction indicated the proteasome pathway is a key target for NCP to regulate gut microbiota and intervene in AD; this pathway was significantly enriched in the AD model group and was a common differential pathway between control vs. model and model vs. NCP groups, suggesting its abnormality is closely related to AD pathology and NCP's regulatory effect. Existing studies [64] confirmed that the UPS, composed of the proteasome pathway, is the core intracellular protein degradation system; its dysfunction leads to the accumulation of abnormally folded proteins (e.g., Aβ, phosphorylated tau), exacerbating AD pathology. Studies [65, 66] also found that protein clearance mechanism failure directly induces Aβ deposition and tau hyperphosphorylation in sporadic AD patient neuronal models. Notably, UPS and autophagy have a close interactive regulatory relationship, jointly constituting the core network for intracellular protein homeostasis. In this study, NCP targets the proteasome pathway by regulating gut microbiota and is speculated to further regulate the UPS‐autophagy interactive network. This echoes the previous metabolomics result that NCP alleviates AD mice's autophagic flux disorders, and Western blot results of autophagy‐related proteins further verify this conclusion, which mutually corroborates the aforementioned metabolomics and gut microbiota findings.

Mechanistically, most existing TCM anti‐AD studies are limited to a single pathological link or pathway [67]. By integrating metabolomics, intestinal flora, and molecular biology technologies, this study reveals NCP's multi‐pathway synergistic anti‐AD mechanism involving “metabolism‐flora‐autophagy”, breaking traditional limitations and providing a typical example for the advantages of “multi‐components, multi‐targets, multi‐pathways”. Although NCP exhibits weaker in vitro AChE inhibitory activity than the clinical drug donepezil (IC_50_: 136.1 vs. 24.3 µm), it exerts satisfactory in vivo anti‐AD effects. As a natural active component derived from *Lycium barbarum* leaves, NCP acts via a multi‐target and multi‐pathway mechanism to ameliorate AD‐related pathologies. Meanwhile, it also shows favorable safety properties, making it a promising candidate for the development of novel natural anti‐AD agents. This study has limitations: first, the D‐galactose combined with AlCl_3_‐induced AD model differs from clinical pathological characteristics, requiring future verification in APP/PS1 transgenic models to improve clinical transformation value; second, the specific interactive molecular mechanisms of the “metabolism‐flora‐autophagy” pathway remain unclear and need further in vitro verification. Future research can focus on exploring core molecular targets of the three‐way interaction, clarifying key signal nodes regulated by NCP, and providing a new direction for precise AD intervention.

## 4 Conclusion

In this study, an innovative technology combining hydrogen peroxide test strip colorimetric sensing with affinity chromatography was constructed, providing an efficient and visual new method for the screening of AChE inhibitors. In vivo experiments confirmed that NCP, an alkaloid from LBL, is a core anti‐AD active component. It can exert in vivo anti‐AD effects by improving cognitive function, protecting hippocampal neurons, inhibiting neuroinflammation, alleviating oxidative stress, and reducing Aβ deposition in AD model mice. Mechanistically, NCP reshapes membrane lipid homeostasis by regulating glycerophospholipid metabolism and linoleic acid metabolism, remodels the intestinal flora structure, and targets the Proteasome pathway, thereby regulating the expression of autophagy‐related proteins (SQSTM1, Beclin‐1, LC3, p‐mTOR/mTOR) to alleviate autophagic flux disorders, forming a multi‐pathway synergistic regulatory network. This study not only enriches the research on the medicinal value of LBL but also provides candidate compounds, action targets, and technical support for the development of anti‐AD natural drugs. In future studies, we will further validate the anti‐AD efficacy of NCP in APP/PS1 transgenic mice. We will also carry out structural modification and optimization to improve its acetylcholinesterase inhibitory activity and blood‐brain barrier permeability, laying a solid foundation for the development of novel anti‐AD drug candidates.

## 5 Experimental Section

### 5.1 Materials and Reagents

AChE, ACh, choline oxidase (ChOx), 3,3’,5,5’‐tetramethylbenzidine (TMB), donepezil, amino‐silica (SiO_2_), allopurinol, glutamic acid, and lysine were procured from Aladdin Biochemical Technology Co., Ltd., Shanghai, China. Horseradish peroxidase (HRP) and D‐galactose were sourced from Macklin Biochemical Technology Co., Ltd., Shanghai, China. Hydrogen peroxide test strips were obtained from Machery‐Nagel GmbH & Co. KG. Acetonitrile (chromatographic grade) was purchased from Sigma‐Aldrich Co. LLC. Methanol (chromatographic grade), BCA Protein Assay Kit and PageRuler Prestained Protein Ladder were acquired from Thermo Fisher Scientific Inc. Phosphate‐buffered saline (PBS, pH = 7.4), Tris‐buffered saline (TBS), and GSH Assay Kit were provided by Wuhan Saiwei Biotechnology Co., Ltd. Kits for Aβ_40_, Aβ_42_, TNF‐α, IL‐1β and IL‐6 were all sourced from Wuhan Elabscience Biotechnology Co., Ltd. Formic acid (chromatographic grade) was purchased from MREDA Technology Inc. Glutaraldehyde was obtained from Jiangsu Pulus Biological Technology Co., Ltd. Chlorogenic acid was acquired from the National Institutes for Food and Drug Control, China. AlCl_3_ was supplied by Tianjin Damao Chemical Reagent Factory. Protein‐free rapid‐blocking solution, PAGE Gel Fast Preparation Kit, Tris‐Glycine‐SDS Running Buffer, and Tris‐Glycine‐SDS Transfer Buffer were all obtained from Shanghai Yaenzyme Biopharmaceutical Technology Co., Ltd. Tween 20 was purchased from Biotopped Life Science. WB Enhanced ECL Luminescent Substrate was procured from Invigentech. PVDF Transfer Membrane was obtained from Immobilon‐P. SDS‐PAGE Loading Buffer was obtained from CW BIO. GAPDH polyclonal antibody, Affinipure goat anti‐rabbit HRP (H+L), Affinipure goat anti‐mouse HRP (H+L), SQSTM1 antibody, LC3 antibody, Beclin‐1 antibody, mTOR antibody, and p‐mTOR antibody were all purchased from Proteintech. All nine types of herbal medicines were sourced from local markets and authenticated in accordance with the Chinese Pharmacopoeia (2025 Edition). All other chemicals were of analytical reagent grade and supplied by local vendors.

### 5.2 Determination of AChE Activity

The experimental procedures were conducted as follows: Six EP tubes were each filled with ACh (400 µL of 500 µg/mL) solution, followed by sequential addition of 400 µL of AChE at varying concentrations (2000, 1000, 500, 250, 125 and 62.5 µg/mL) to each tube; after 30 min of incubation, 200 µL of a ChOx (50 µg/mL) solution was added and incubated for another 30 min. The activity of AChE was assessed using hydrogen peroxide test strips, which exhibited a blue color in response to the presence of hydrogen peroxide. The intensity of the blue color correlated with the concentration of AChE, with higher concentrations resulting in greater hydrogen peroxide production and a deeper blue hue on the test strip. For the colorimetric‐based quantitative analysis, the above steps remained unchanged except for the addition of 200 µL of an HRP (5 µg/mL) and 400 µL of a TMB (1 mg/mL) solution alongside ChOx. After a 30‐min reaction, 200 µL of a TMB chromogenic stop solution was added, and the absorbance was measured at 450 nm. An AChE standard curve was then constructed, plotting concentration on the abscissa and absorbance on the ordinate.

### 5.3 Preparation of AChE@SiO_2_ to Prepare an Affinity Chromatography Carrier

50 mg of SiO_2_ was dispersed in 4 mL of PBS, and glutaraldehyde was added; the mixture was shaken for 2 h for activation and washed thrice with deionized water. AChE was then introduced for shaking for 4 h to realize enzyme immobilization on the SiO_2_ surface. Next, 1 g of lysine dissolved in 10 mL of PBS was added to the system, shaken for 2 h for reaction, centrifuged, and washed thrice with deionized water to yield the AChE@SiO_2_ affinity chromatography carrier. Finally, the carrier was resuspended in 5 mL of PBS, aliquoted, and stored at −20°C for later use.

### 5.4 Characterization of AChE@SiO_2_

Scanning electron microscopy (SEM) and atomic force microscopy (AFM) were combined to characterize the surface morphologies of SiO_2_ and AChE@SiO_2_. X‐ray photoelectron spectroscopy (XPS) analyzed their chemical compositions. Thermogravimetric analysis (TGA) via a simultaneous thermal analyzer confirmed AChE immobilization on SiO_2_ and the differences in their thermal behavior, while Fourier transform infrared spectroscopy (FTIR) further verified the immobilization.

### 5.5 Establishment and Evaluation of AChE Inhibitor Screening Model

The present study developed a dual model coupling a biosensor and affinity chromatography to identify AChE inhibitors. To validate the specificity and sensitivity of the model, both positive (donepezil) and negative (glutamic acid, lysine, and allopurinol) drugs were employed. For the biosensor section, 200 µL of either positive or negative solutions at varying concentrations were incubated with AChE (400 µL of a 500 µg/mL) solution for 30 min. Subsequent procedures followed those outlined in section “5.2”. The color changes on the test strips were recorded using a smartphone. For the affinity chromatography section, positive or negative solutions (200 µL of 50 µg/mL) were combined with AChE@SiO_2_ (400 µL of a 500 µg/mL) carrier. The mixture was shaken for 30 min, centrifuged, and the supernatant was collected as the unadsorbed solution. The residual solid was then treated with 200 µL of 50% acetonitrile, shaken for an additional 30 min, centrifuged, and the supernatant was collected as the eluted solution. HPLC was used to analyze the initial, unadsorbed, and eluted solutions from each group. The adsorption rate was calculated using the formula: Adsorption rate = (1 – Peak area of drug in unadsorbed solution / Peak area of drug in initial solution) × 100%.

### 5.6 Screening of Active Components From Herbal Medicines

Following validation with both positive and negative controls, the model was subsequently employed to identify potential AChE‐inhibiting active components in herbal medicines. A total of nine herbal samples were used, each material being crushed and sieved; 2 g of each sieved sample underwent ultrasonic extraction using 50 mL of ethanol for 30 min, followed by filtration and drying to obtain crude extracts. Then, AChE (400 µL of a 500 µg/mL) solution was mixed with 200 µL of each crude extract, reacted for 30 min, and processed according to the procedure outlined in section “5.2”. The color changes of the test strips were recorded using a smartphone. According to the principle, herbal medicines exhibiting an AChE inhibitory effect resulted in test strip colors that were either non‐blue or displayed a shallow blue density. Herbal medicine extracts demonstrating these characteristics, indicative of potential AChE inhibitory components, were selected for further affinity identification and separation, following the protocol described in Section 5.5. HPLC and UPLC‐MS were applied to analyze the initial, unadsorbed, and eluted solutions, respectively.

### 5.7 HPLC‐DAD and UHPLC‐MS Analysis

Chromatographic analysis was performed using a Shimadzu 2030C 3D HPLC with a C18 column (4.6 × 150 mm, 3.5 µm) at 35°C, a 320 nm detection wavelength, a 10 µL injection volume, and a 0.8 mL/min flow rate. Samples were pre‐filtered with a 0.22 µm PVDF membrane. The mobile phases were 0.1% phosphoric acid (A) and methanol (B), with a gradient elution as follows: 98% A (0 min); 88% A (3 min); 85% A (12 min); 77% A (20 min); 67% A (35 min); 55% A (45 min); 98% A (48–50 min). Analytes were identified by retention times and spectra comparison with standards.

For potential bioactive compounds, an Agilent 6546 QTOF‐MS with a 1260 Infinity HPLC was used, employing a Symmetry C18 column (2.1 mm × 50 mm, 3.5 µm) and mobile phases of 0.1% formic acid (A) and 0.1% formic acid in acetonitrile (B). The gradient elution was programmed as follows: 98% A (0–2 min), 90% A (8 min), 88% A (15 min), 75% A (18 min), 55% A (28 min), 45% A (35 min), 10% A (50 min), and 98% A (55–60 min) at a flow rate of 0.2 mL/min. Mass spectrometry was conducted in both positive and negative ion modes with these settings: drying gas at 320°C and 8 L/min, nebulizer at 35 psi, sheath gas at 350°C and 11 L/min, and a mass range of 50–3000 m/z. Each analysis included precise mass calibration [68, 69].

### 5.8 Determination of IC_50_ of AChE‐Inhibitory Active Components

PBS was used to prepare serial solutions of identified potential AChE inhibitor compounds at concentrations of (5, 10, 15, 20, 25, 30, 35, 40, 50, 60, 100, 200, and 400 µg/mL). Subsequent procedures followed the protocol described in section “5.2”. Absorbance measurements at 450 nm were obtained using an ultraviolet–visible spectrophotometer, and the half‐maximal inhibitory concentration (IC_50_) values for AChE inhibition were calculated for each compound.

### 5.9 Molecular Docking, Molecular Dynamics Simulation, and Density Functional Theory (DFT)

To clarify the binding modes and conformational stability of complexes formed by the three selected monomers and AChE, comprehensive molecular docking and molecular dynamics simulations were performed. The AChE crystal structure (PDB ID: 1eve) was retrieved from the Protein Data Bank and preprocessed using the Protein Preparation Wizard in Discovery Studio. The preprocessing included protein pretreatment, native ligand state restoration, hydrogen bond assignment optimization, and energy minimization. The binding site was defined based on the ligand‐binding pocket in the original co‐crystal structure. Molecular dynamics simulations of the complexes were conducted using Schrödinger 2024 Linux with a 1000 ns simulation duration. Finally, DFT calculations were performed using the Gaussian 09 D.01 Linux program to systematically investigate the electronic structure information and weak interaction characteristics.

### 5.10 Animal Experiment

Forty‐eight SPF‐grade ICR male mice (20–30 g) from Ningxia Medical University's Experimental Animal Center (License No.: SYXK(Ning)2025‐0001) were housed in an SPF barrier environment with a 12‐h light/dark cycle and had free access to food and water. All animal experiments in this study were approved by the Animal Ethics Committee (Ethics No.: 2024–052) and strictly performed in accordance with the ARRIVE guidelines and the 3Rs principles (Replacement, Reduction, Refinement) for animal research. Before experiments, all experimental animals were randomly assigned to different groups. For behavioral assessment, the evaluators were kept blinded to the group information to avoid subjective bias. Meanwhile, well‐defined humane endpoints were applied throughout the study to ensure animal welfare. We adopted the D‐galactose/AlCl_3_ combined model to establish AD mice. This model can stably simulate sporadic AD and presents typical pathological phenotypes consistent with our research objectives. Transgenic models, including APP/PS1 and 5xFAD, are primarily used for familial AD research with different pathological characteristics. In addition, this chemical induction model has the merits of low cost, short experimental cycle, and high stability, which is suitable for pharmacodynamic evaluation of natural products. An AD model was established through subcutaneous injection of D‐galactose (200 mg/kg) combined with intragastric administration of AlCl_3_ (150 mg/kg) over a period of 10 consecutive weeks, during which drug administration was performed concurrently. Specifically, following 3 days of acclimatization, mice were randomized assigned based on body weight into 5 groups (n = 8 each): a control group, an AD model group, a positive (donepezil, 5 mg/kg) group, and two groups receiving the AChE inhibitor NCP at low (NCP‐L, 10 mg/kg/d) and high (NCP‐H, 25 mg/kg/d) doses. Then, the AD model was induced using the above method, and the drug was orally administered simultaneously. All drugs were dissolved in saline and administered intragastrically at 10 mL/kg, with the control and model groups receiving an equal volume of normal saline. After 10 weeks of treatment, the behavioral tests were conducted sequentially. After all behavioral tests were completed, fresh fecal samples were collected immediately. Each mouse was placed in a sterile empty cage without bedding materials for 5–10 min to obtain fresh feces. The naturally excreted fecal pellets were quickly transferred into sterile cryogenic vials using sterile tweezers. All samples were immediately frozen in liquid nitrogen and then stored at −80°C until subsequent 16S rRNA gene sequencing analysis. To avoid cross‐contamination, cages and tweezers were disinfected thoroughly between individual mice. Then, mice were euthanized via cervical dislocation, blood samples were collected, stood for 2 h, centrifuged at 2000 rpm for 10 min, and the supernatant was further centrifuged at 13 000 rpm for 10 min; the final supernatant was stored at −80°C for further analysis; brains were harvested on ice, snap‐frozen in liquid nitrogen, and stored at −80°C for subsequent assays.

### 5.11 Y‐Maze Test

The Y‐maze test was performed to assess the effect of the identified AChE inhibitor, NCP, on working memory in AD mice. The maze consisted of three identical white‐bottomed rectangular arms intersecting at 120°, labeled A, B, and C at their distal ends. Each mouse was placed in the central triangle and allowed 8 min of free exploration. An effective arm entry was defined as the mouse's body (excluding the tail) mostly entering an arm; arm entry sequences were recorded to calculate the spontaneous alternation rate.

### 5.12 Novel Object Recognition Test

This test assessed mice's learning and memory abilities by their innate novel environment exploration tendency, with all operations performed in a clean, odorless cage across three phases. Acclimation phase: mice were placed in an empty cage for 5 min of free activity, then returned to the cage. Training phase: Two identical objects (A1, A2) were placed in the cage; mice explored for 5 min, then rested in the cage for 5 min. Testing phase: A2 was replaced with a shape‐ and size‐differentiated Object B, and mice explored for 5 min. An exploration event was defined as head contact with an object, and the touch frequency of each object was recorded.

### 5.13 Morris Water Maze Test

The experimental apparatus included a circular water tank (0.5 m diameter, black inner walls), a 30 cm‐high black cylindrical platform, and supporting camera equipment. The water level was 1 cm above the platform, and the tank was divided equally into 4 quadrants. Mice underwent 1 day of environmental acclimation before the formal experiment, which comprised two parts: place navigation and spatial probe tests. For the place navigation test, the platform was placed in the center of Quadrant 1. Mice were sequentially placed into the water from the back wall of each quadrant, with a 90‐s time limit to locate the platform. Mice that found the platform rested on it for 10 s before the next trial; those that failed were assigned a latency of 90 s. This test lasted 5 consecutive days. One day later, the spatial probe test was performed: the platform was removed, mice were placed into the water from any quadrant wall, and the number of crossings over the original platform location within 60 s was recorded. All behavioral data were analyzed via SPSS 26.0.

### 5.14 ELISA Assay of Mouse Brain Tissue

Mouse brain tissues were homogenized thoroughly using a tissue grinder to prepare brain tissue homogenates. The homogenates were centrifuged at 5000 xg/min for 10 min at 4°C, and the supernatants were collected. According to the manufacturer's instructions, commercial kits were used to detect the levels of Aβ_40_, Aβ_42_, TNF‐α, IL‐1β, IL‐6, and GSH.

### 5.15 HE and Nissl Staining of Hippocampal Region in Brain Tissue

Brain tissues were fixed in 4% paraformaldehyde for 24 h, followed by paraffin embedding, sectioning, xylene deparaffinization, and gradient ethanol dehydration. Subsequently, HE staining and Nissl staining were performed. Pathological observation and photography of the hippocampal region of the brain tissue were carried out under a microscope.

### 5.16 Untargeted Metabolomics

Mouse serum samples were thawed; 50 µL of each sample was pipetted into an EP tube, followed by the addition of 200 µL of pre‐cooled acetonitrile solution containing the internal standard, astragaloside IV. The mixture was vortexed for 30 s, incubated at 4°C for complete protein precipitation, and centrifuged at 13 000 rpm at 4°C for 10 min using a low‐temperature high‐speed centrifuge (repeated twice). The supernatant was collected for injection analysis. Chromatographic conditions:Waters Symmetry C18 column (2.1 mm × 100 mm, 3.5 µm); column temperature 30°C; flow rate (0.2 mL/min); injection volume (1 µL); mobile phases A (0.1% formic acid aqueous solution) and B (0.1% formic acid in acetonitrile); gradient elution program: 98% A (0 min); 45% A (5 min); 35% A (10 min); 25% A (15 min); 15% A (20–23 min); 10% A (25–27 min); 5% A (30 min); 98% A (33–35 min). Mass spectrometry conditions: Electrospray ionization (ESI) dual‐spray ion source, data collected in positive/negative ion modes with Auto MS/MS scanning; parameters:drying gas flow (8 L/min), drying gas temp 320°C, capillary voltage 3500 V, nebulizer pressure 35 psi, sheath gas temp 350°C, sheath gas flow (11 L/min), nozzle voltage 1000 V, skimmer 65 V, fragmentor 130 V, collision energy 30 eV, scanning range 50–3000 m/z. LC‐MS data in.d format were converted to mzML format via ProteoWizard, then imported into Progenesis QI 2.3 for preprocessing (peak detection, extraction, alignment, integration) to generate a three‐dimensional data matrix.

### 5.17 Gut Microbiota

Genomic DNA extracted from gut microbiota was quality‐checked by agarose gel electrophoresis (integrity) and Nanodrop 2000 (concentration, total amount, purity). High‐fidelity PCR amplification of target regions was performed on qualified samples with three replicates; amplified products were mixed and purified. Specific tags were introduced via PCR with Index‐containing primers, and the primary library was obtained after purification. After preliminary and Qubit‐accurate quantification, samples were pooled at an equimolar ratio. The pooled library was analyzed for insert size and concentration using an Agilent 2100, followed by paired‐end sequencing on the Illumina Novaseq platform for subsequent bioinformatics analysis.

### 5.18 Western Blot

Mouse brain tissues were homogenized, and total proteins were extracted with lysis buffer containing protease inhibitors. Protein concentration was quantified via the BCA method. Samples were boiled at 100°C for 8 min, cooled, and 40 µg of protein per sample was loaded onto a 7.5%, 10%, or 12.5% SDS‐PAGE gel. Electrophoresis was run at 60 V for 20 min, then 120 V for 1 h until the bromophenol blue indicator reached the gel bottom. Proteins were transferred onto a PVDF membrane at 360 mA for 1.5 h. The membrane was blocked at room temperature for 30 min, washed thrice with TBST buffer (5 min each), incubated with primary antibodies (mTOR, 1:5000; p‐mTOR, 1:1000; SQSTM1, 1:500; Beclin‐1, 1:1000; LC3, 1:2500) at 4°C overnight, re‐washed, incubated with secondary antibodies (HRP, 1:5000) for 1 h, and washed again. Enhanced chemiluminescence was used for visualization, and band images were analyzed via ImageJ.

## Author Contributions

**Yuping Sa**: Writing – original draft, Investigation, Methodology, Data curation; **Hui Yuan**: Validation, Methodology, Data curation; **Jingui Ma**: Validation, Methodology, Data curation; **Zhigang Yang**: Methodology and data analysis; **Mei Wang**: Validation, Data curation; **Weibiao Wang**: Validation, Methodology; **Lingling Yang**: Data curation, Investigation; **Fen Ma**: Methodology, Investigation; **Weiman Zhang**: Data curation, Methodology; **Gidion Wilson**: Revision; **Guoning Chen**: Project administration, Conceptualization; **Xueqin Ma**: Project administration, Conceptualization.

## Conflicts of Interest

The authors have no conflicts of interest.

## Supporting information




**Supporting File 1**: advs76863‐sup‐0001‐SuppMat.docx

## Data Availability

The data that support the findings of this study are available from the corresponding author upon reasonable request.
