# Supplementary material for: Innovative Test Strip‐Based Colorimetric Sensors Integrated With Affinity Chromatography: Acetylcholinesterase Inhibitor Screening Breakthrough in Lycium Barbarum Leaves
Source: Adv Sci (Weinh). 2026 Jul 29:e76863. Online ahead of print. doi: 10.1002/advs.76863 (PMC13418505; doi:10.1002/advs.76863)
Supplement: Supplementary file 1 — Supporting File 1: advs76863‐sup‐0001‐SuppMat.docx [file ADVS-9999-e76863-s001.docx]

**Innovative test strip-based colorimetric sensors integrated with affinity chromatography: Acetylcholinesterase inhibitor screening breakthrough in *Lycium barbarum* leaves**

Yuping Sa ^a, b, 1^, Hui Yuan ^a, b, 1^, Jingui Ma ^a,b, 1^, Zhigang Yang ^a, b^, Mei Wang ^a,b^, Weibiao Wang ^a, b^, Lingling Yang ^a, b^, Fen Ma ^a, b^, Weiman Zhang ^a, b^, Gidion Wilson ^a, b^, Guoning Chen ^a, b, *^, Xueqin Ma ^a, b, *^

^a^ School of Pharmacy, Ningxia Medical University, 1160 Shenli Street, Yinchuan, 750004, China

^b^ Key Laboratory of Protection, Development and Utilization of Medicinal Resources in Liupanshan Area, Ministry of Education, College of Pharmacy, Ningxia Medical University, 1160 Shenli Street, Yinchuan, 750004, China

^1^ The authors contributed equally to this work

^*^Correspondence authors: Tel/Fax: +86 09516880693, E-mail addresses: maxueqin217@126.com (Xueqin Ma); nycgn2022@163.com (Guoning Chen)


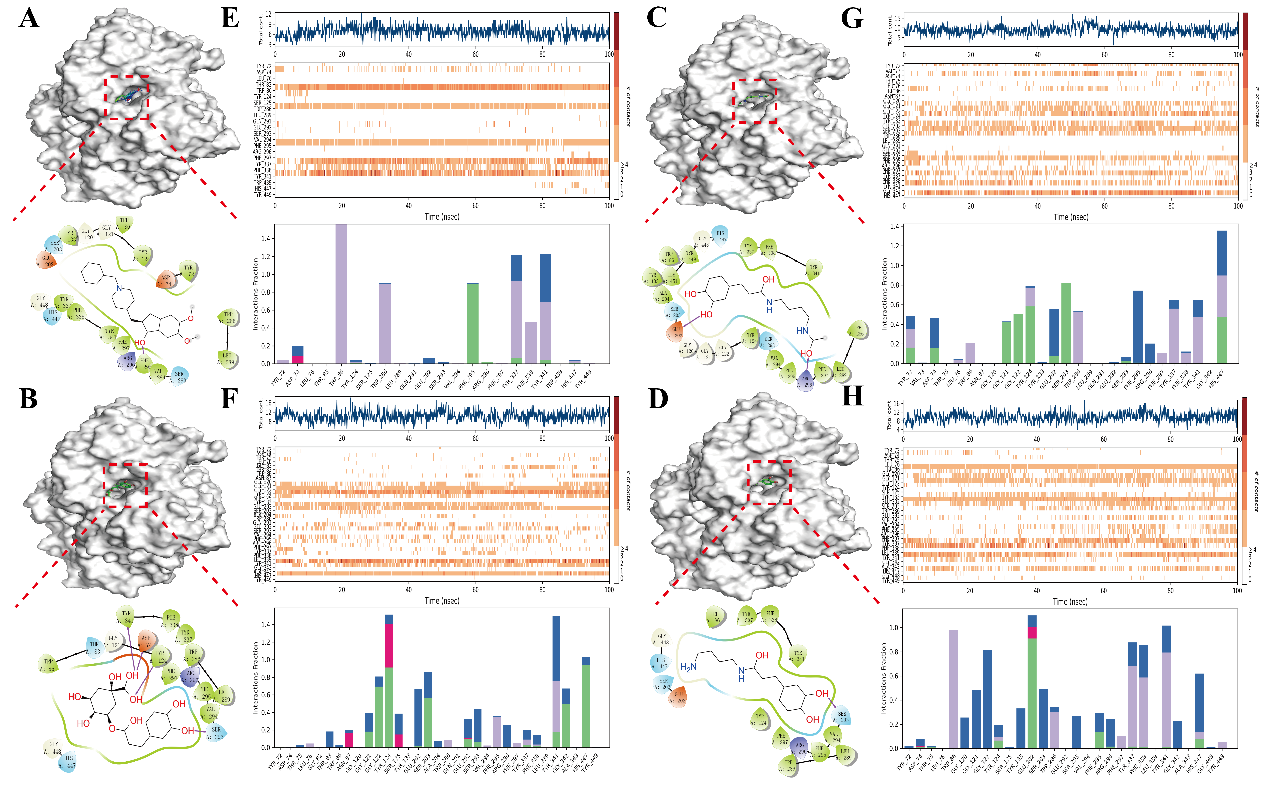


**Supplementary Figure 1** (**A**) donepezil of molecular docking; (**B**) Chlorogenic acid of molecular docking; (**C**)NANCP of molecular docking; (**D**) NCP of molecular docking; (**E-H**) Hydrogen bond occupancy timeline diagrams and ligand-receptor binding interface interaction analysis diagrams of donepezil, Chlorogenic acid, NANCP, and NCP.


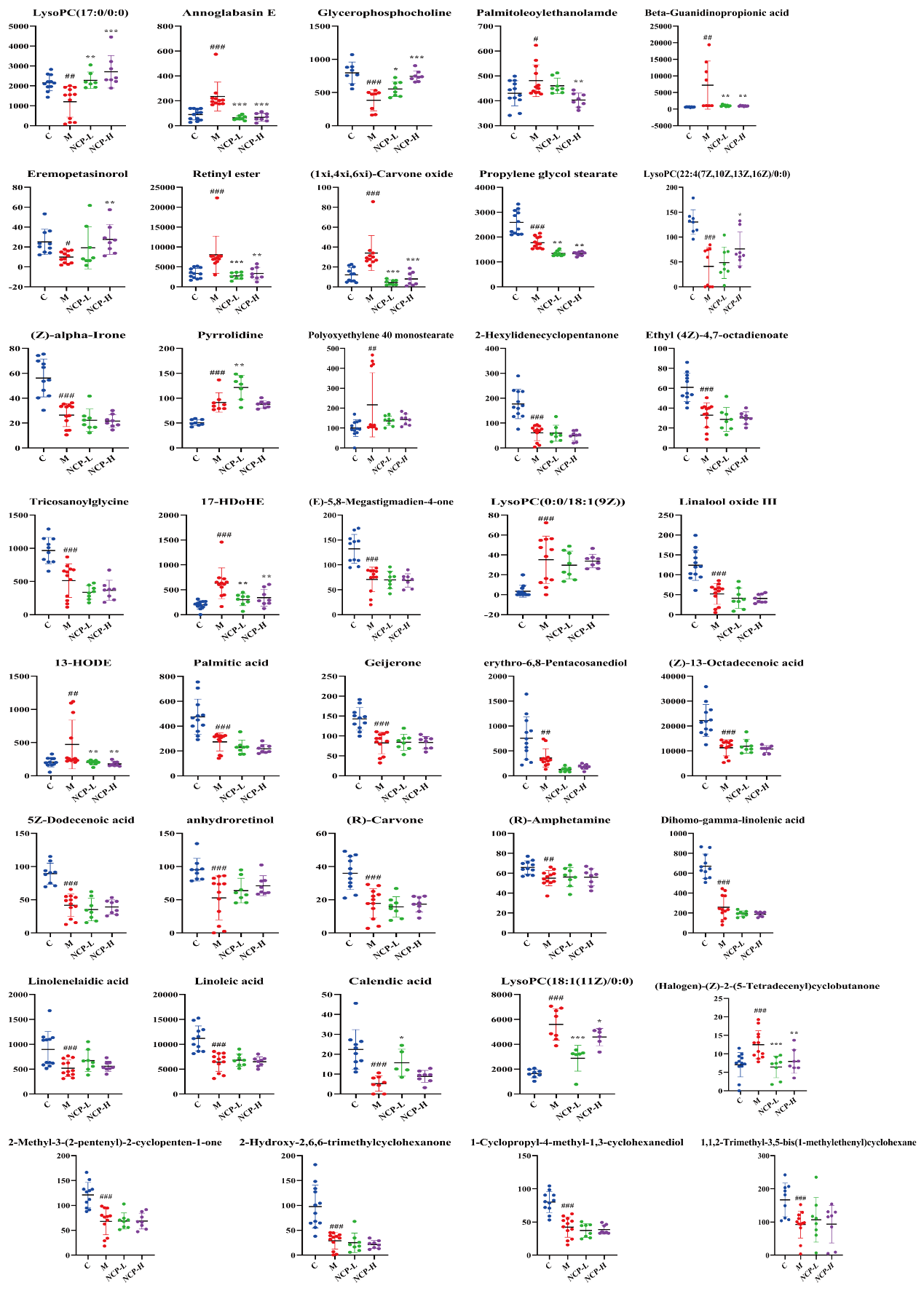


**Supplementary Figure 2** Metabolite point plots in the positive ion modes.


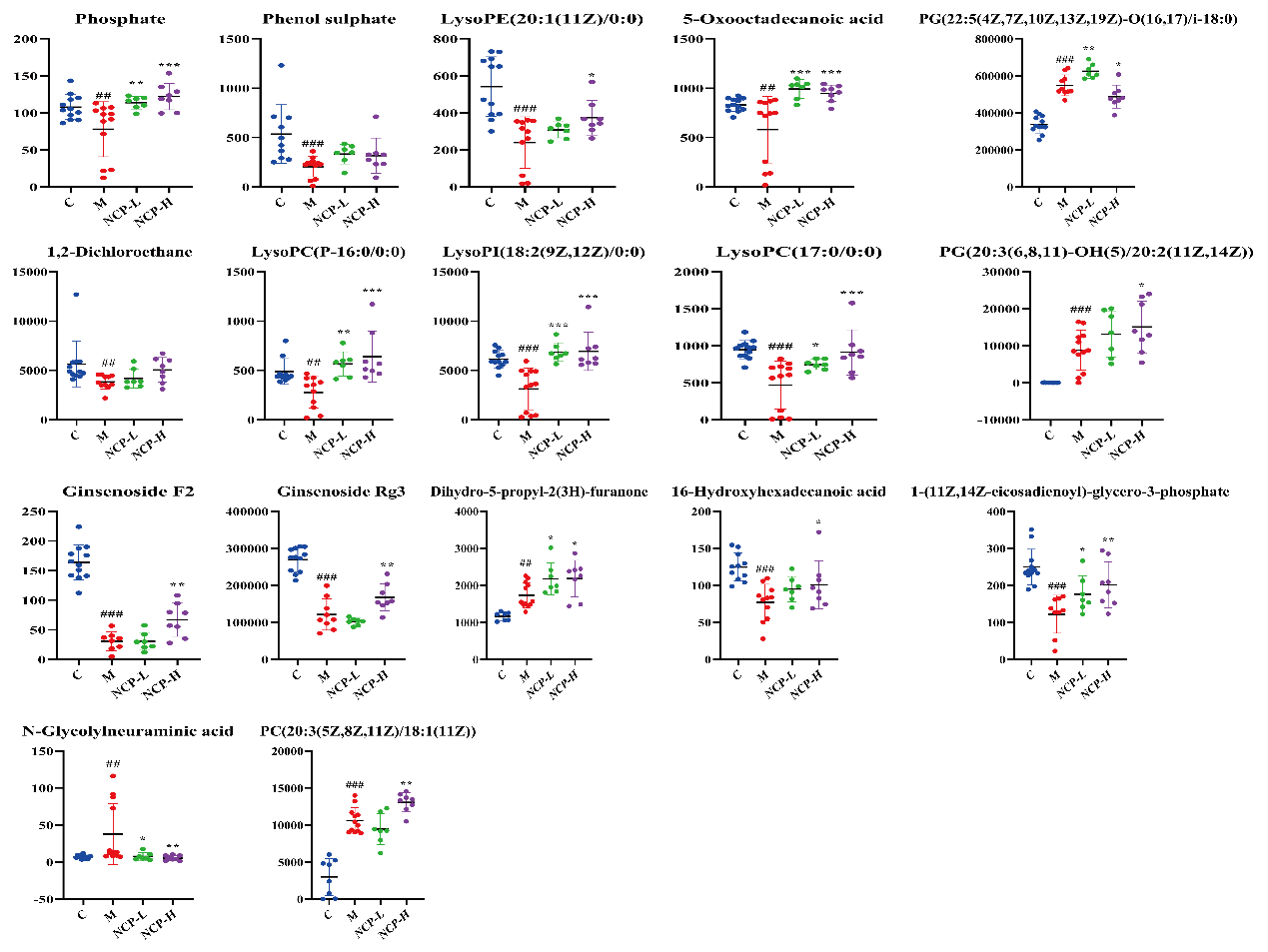


**Supplementary Figure 3** Metabolite point plots in the negative ion modes.


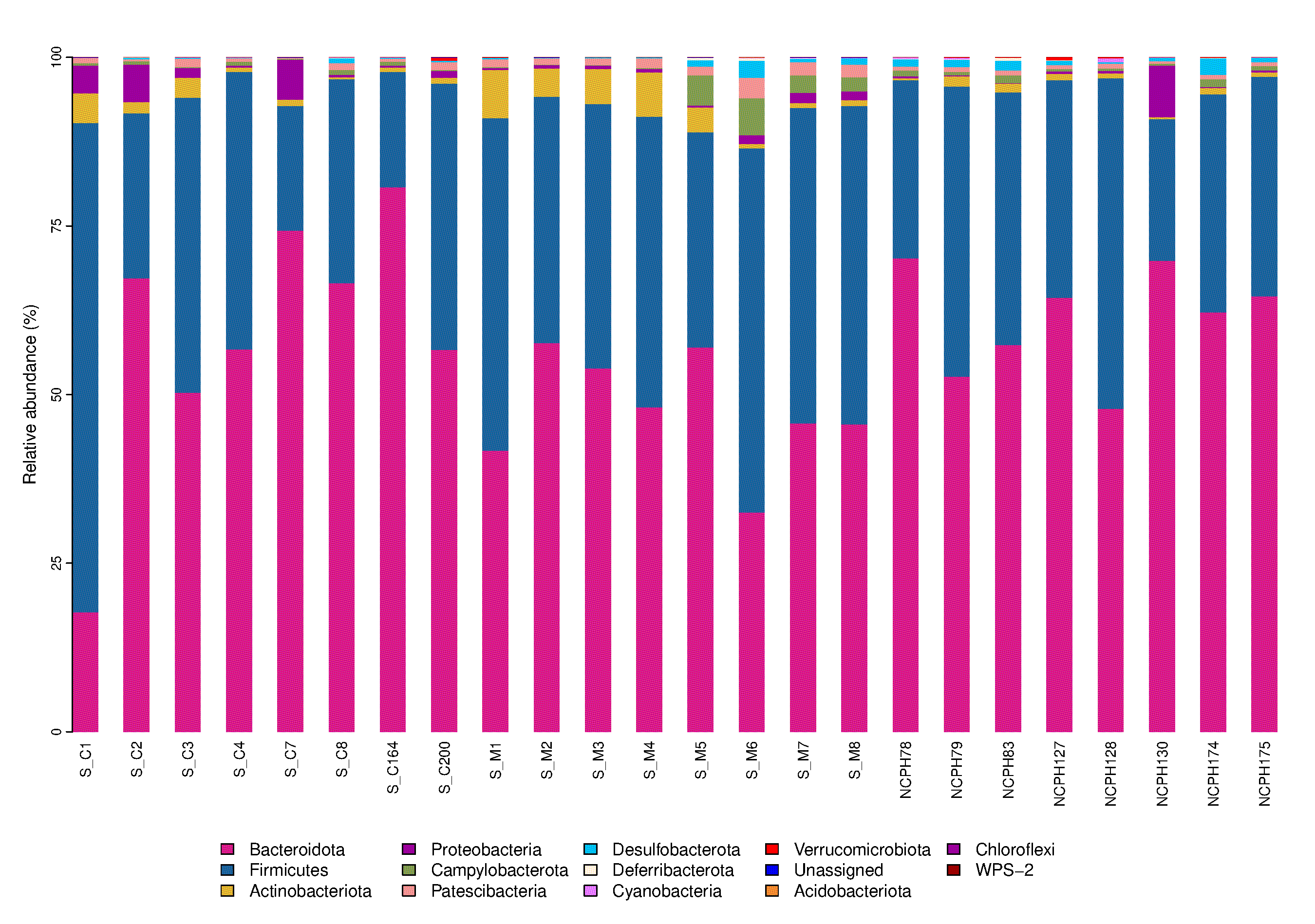


**Supplementary Figure 4** Bar chart of community structure analysis at the phylum level.


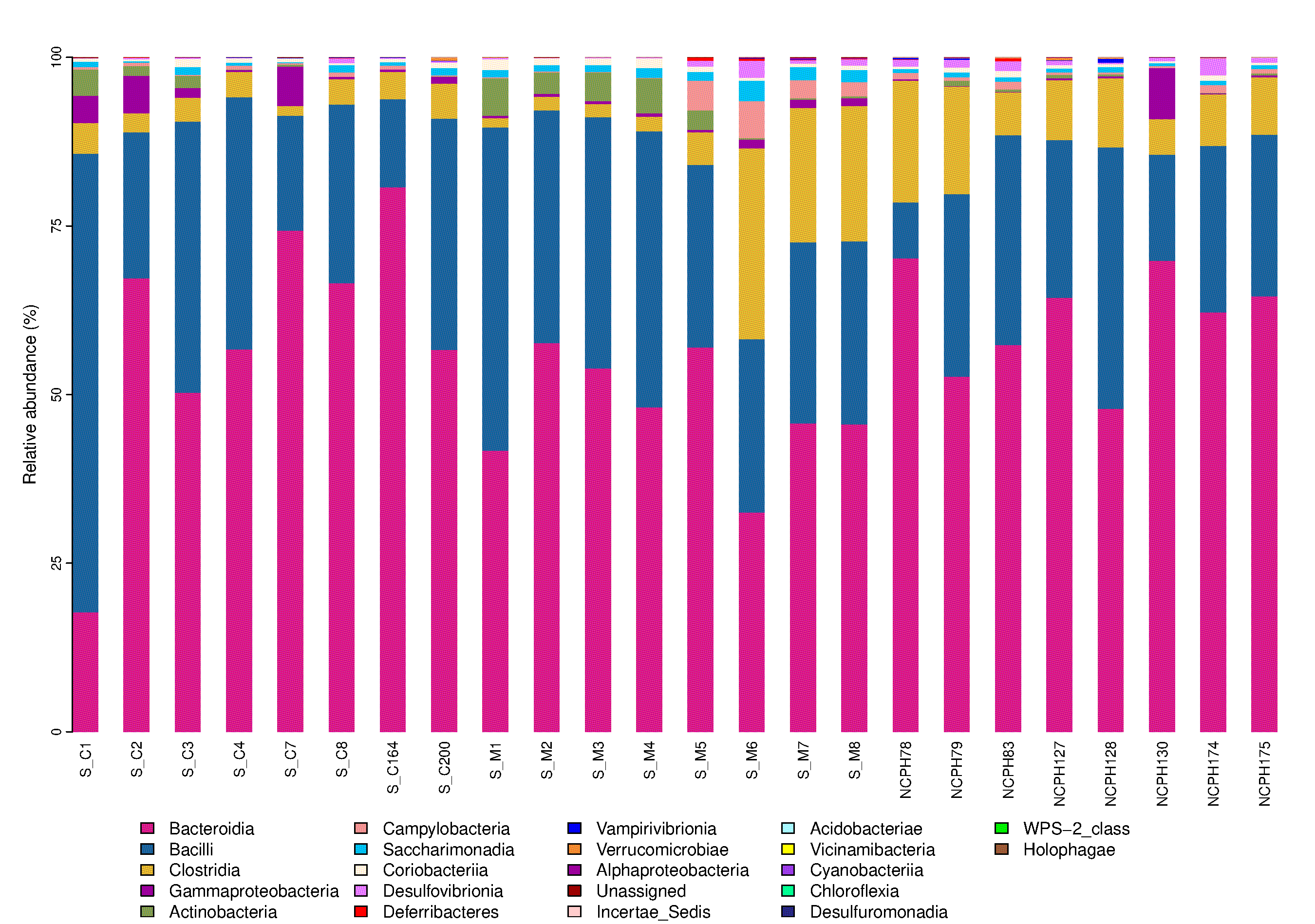


**Supplementary Figure 5** Bar chart of community structure analysis at the class level.


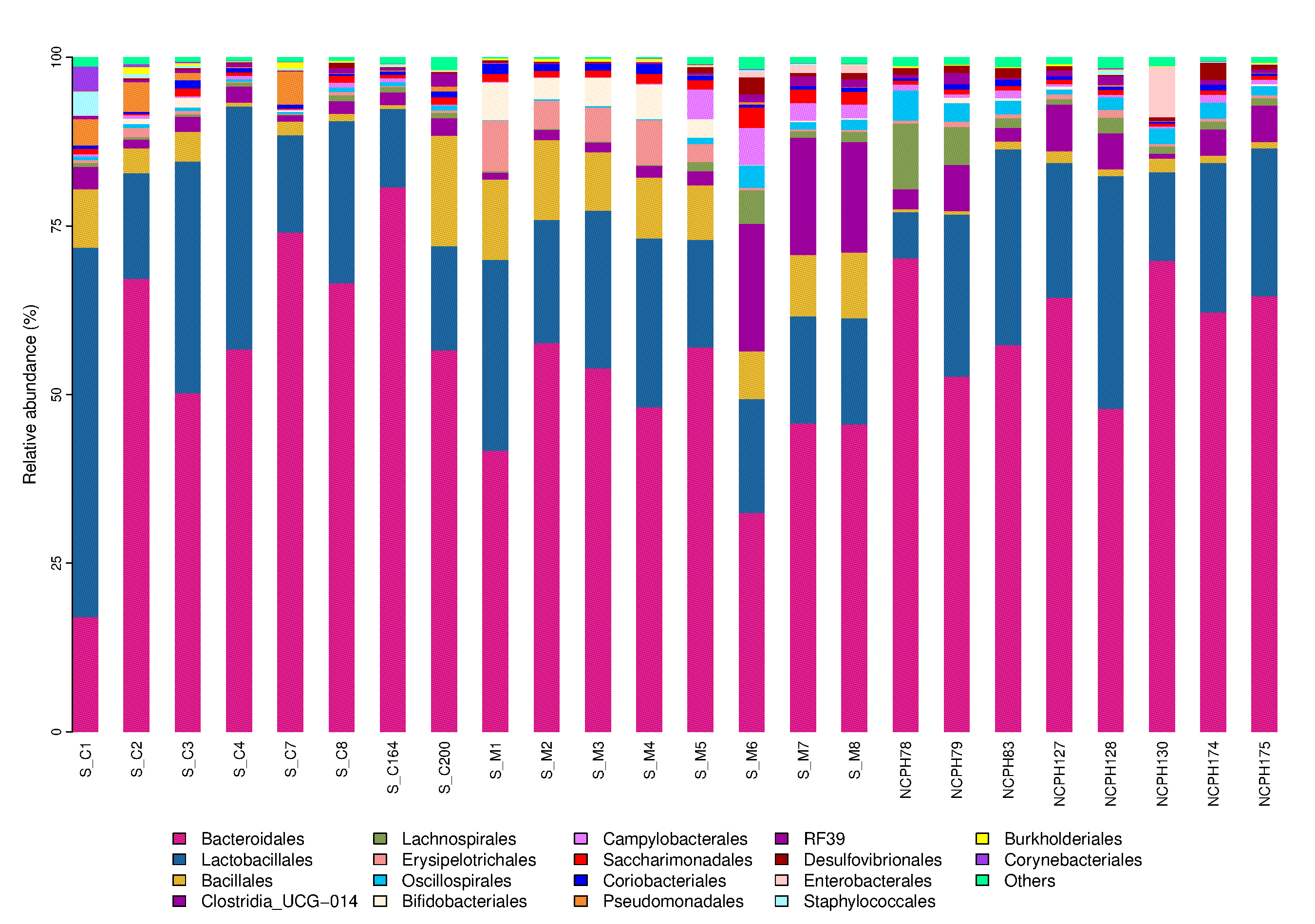


**Supplementary Figure 6** Bar chart of community structure analysis at the order level.


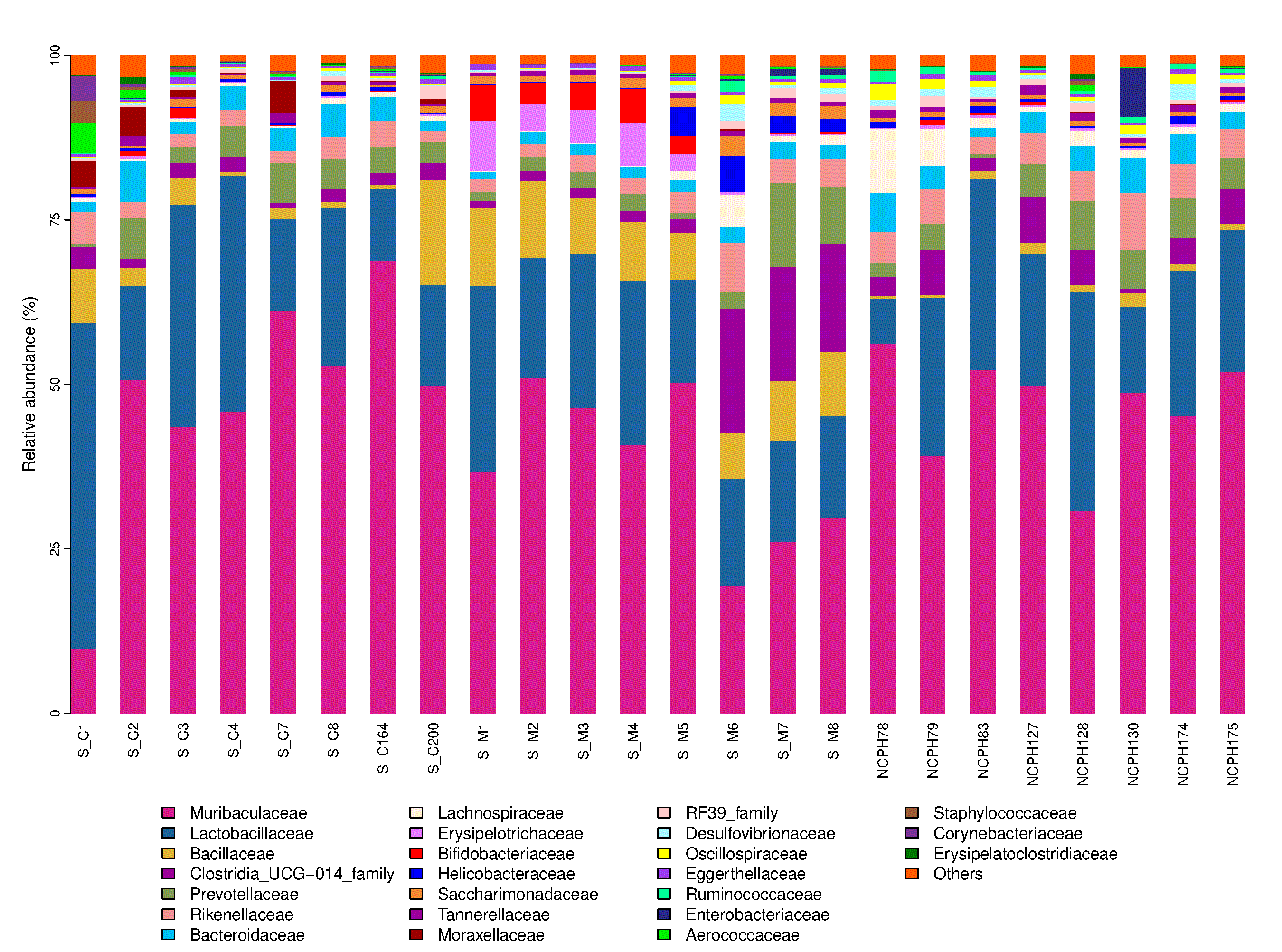


**Supplementary Figure 7** Bar chart of community structure analysis at the family level.


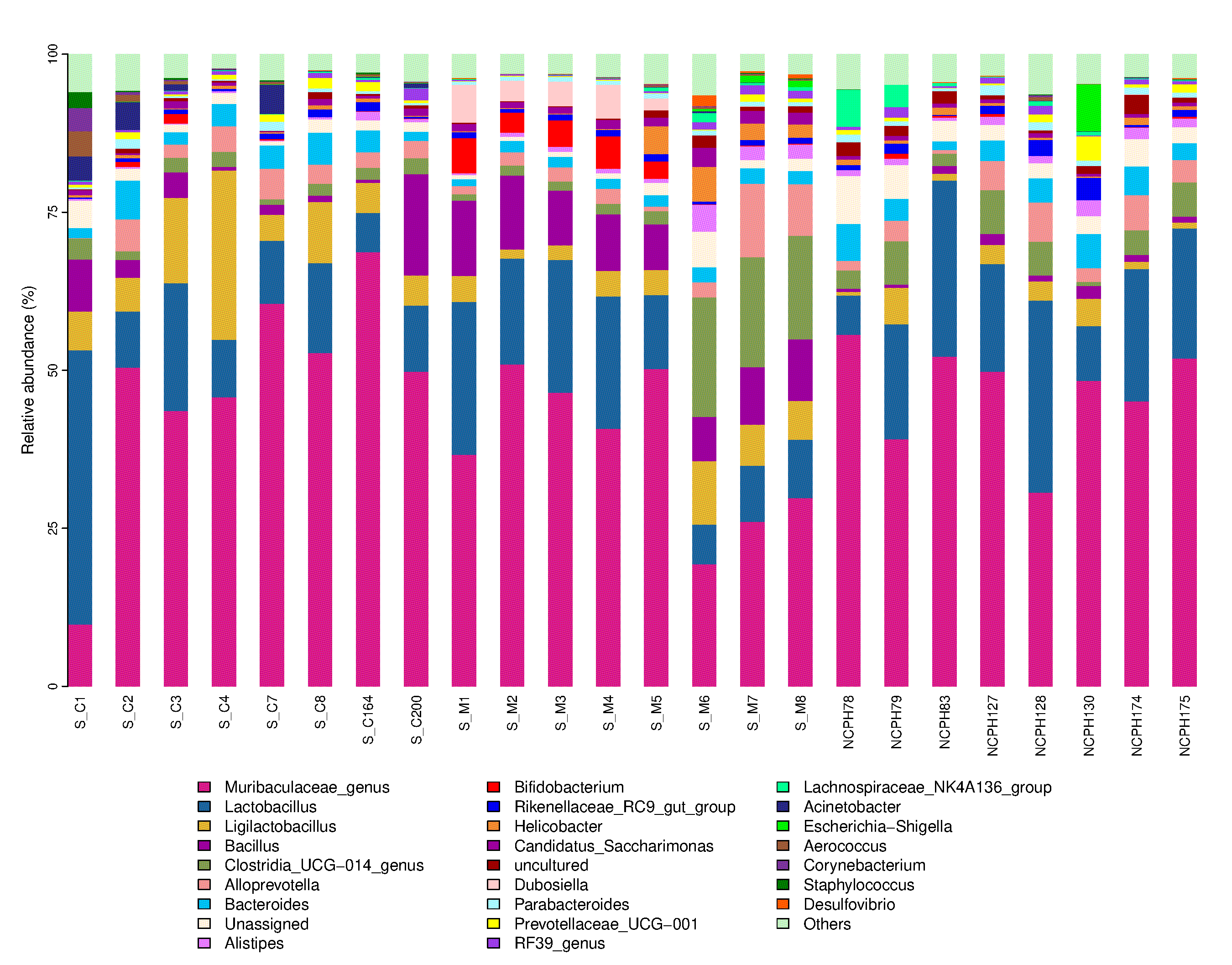


**Supplementary Figure 8** Bar chart of community structure analysis at the genus level.


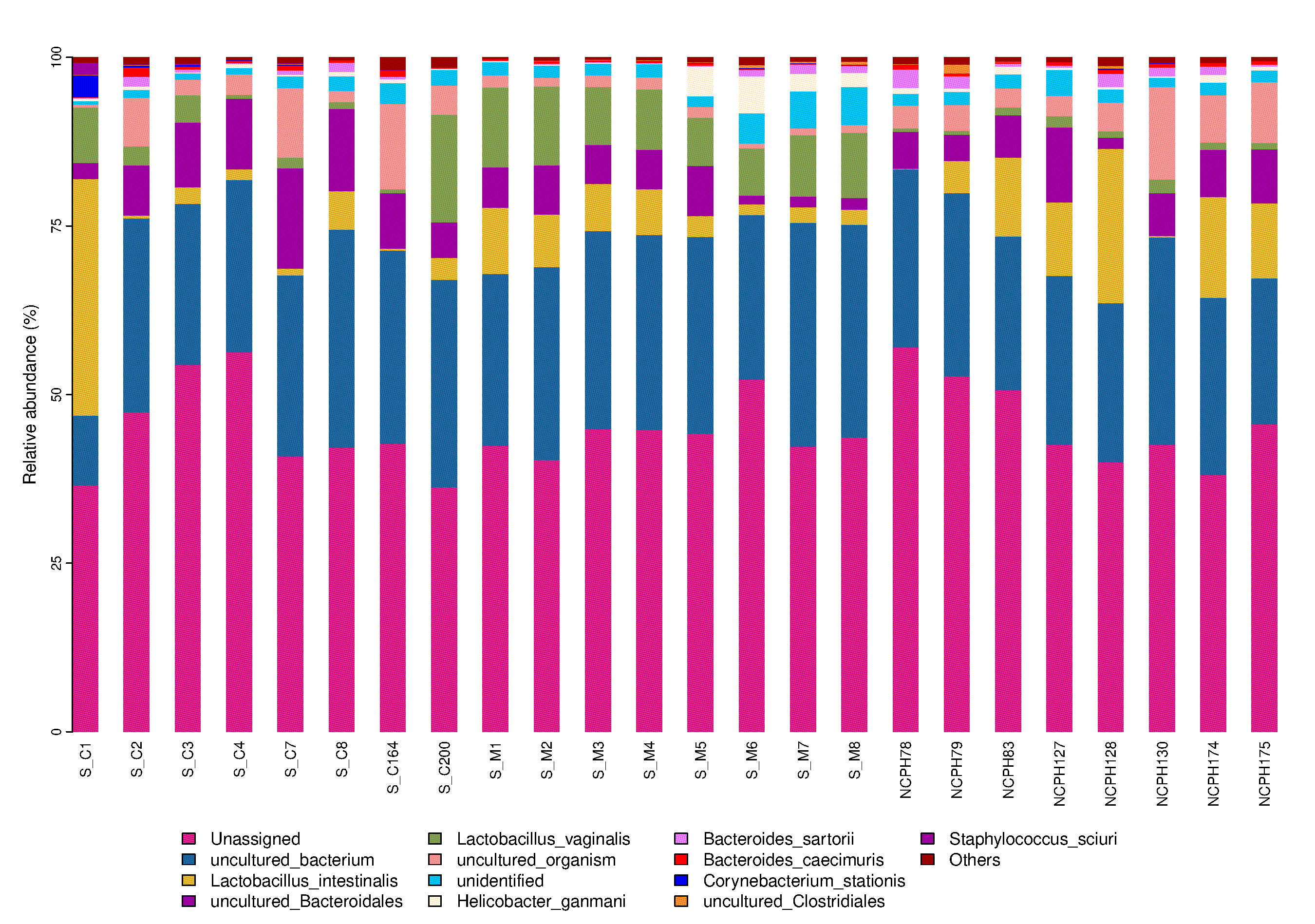


**Supplementary Figure 9** Bar chart of community structure analysis at the species level.


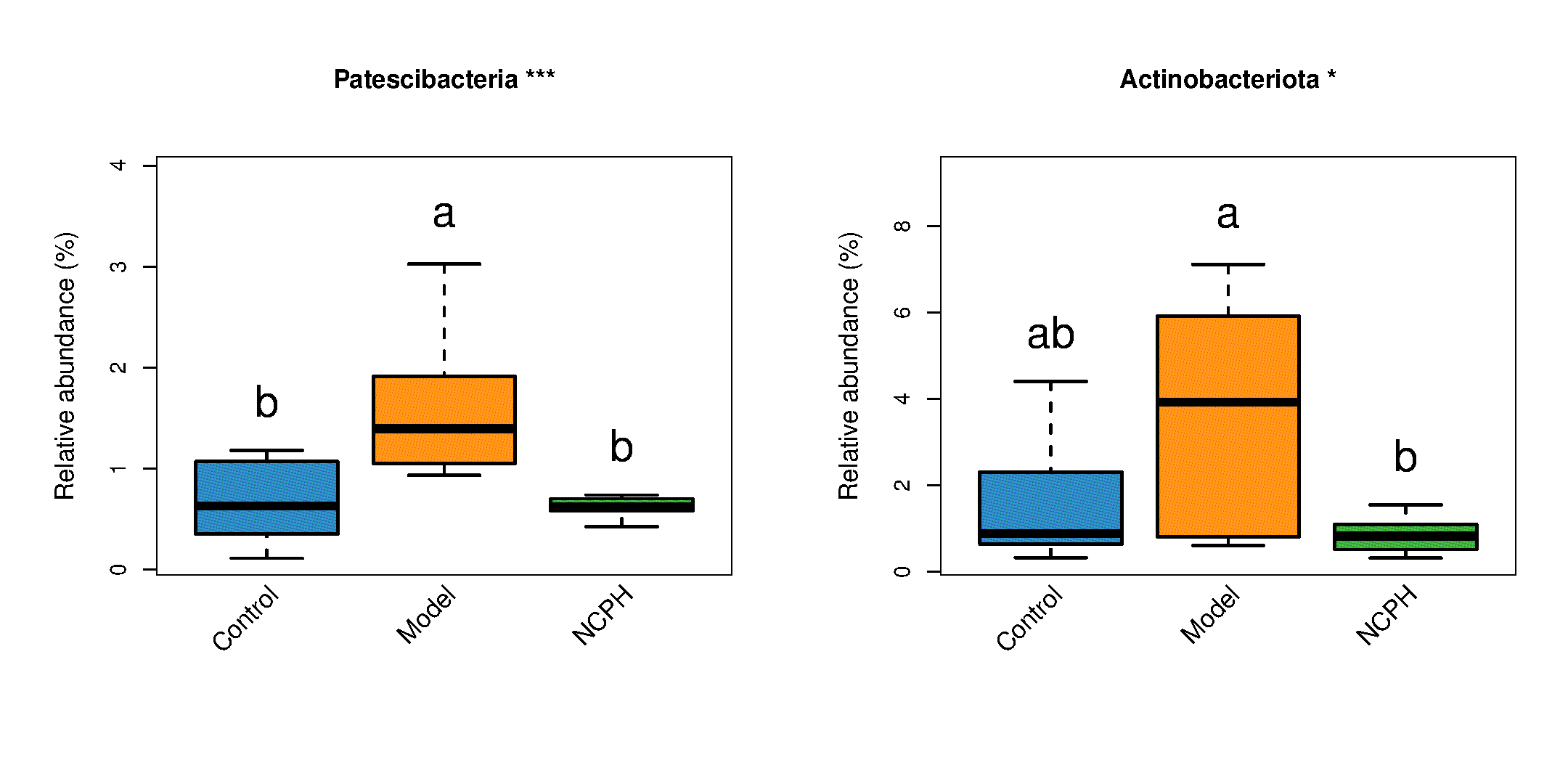


**Supplementary Figure 10** Box plot of community structure analysis at the phylum level.


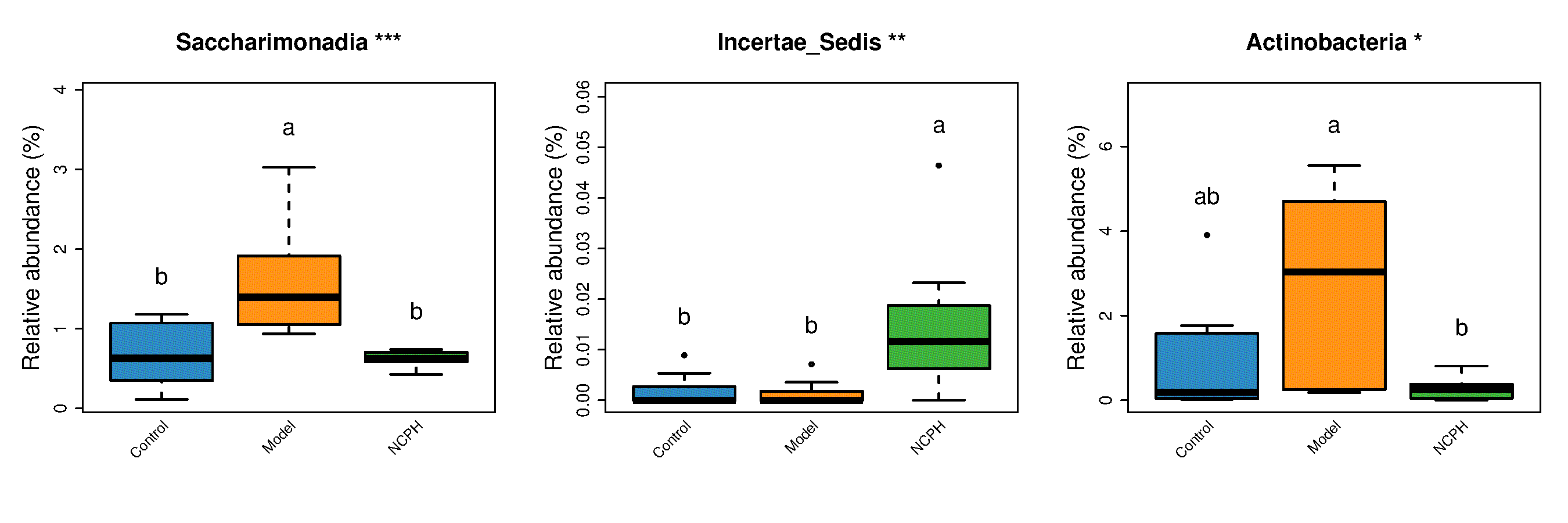


**Supplementary Figure 11** Box plot of community structure analysis at the class level.


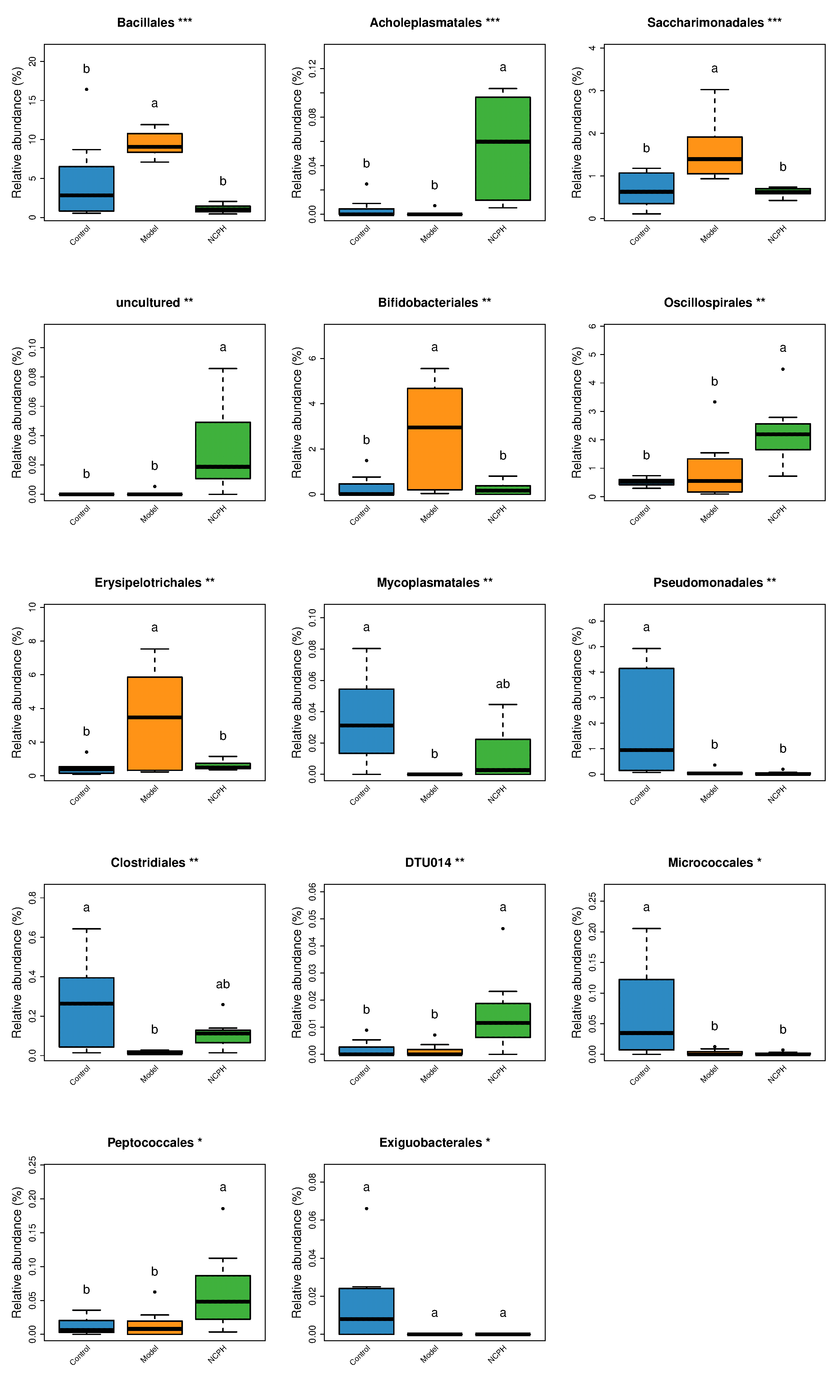


**Supplementary Figure 12** Box plot of community structure analysis at the order level.


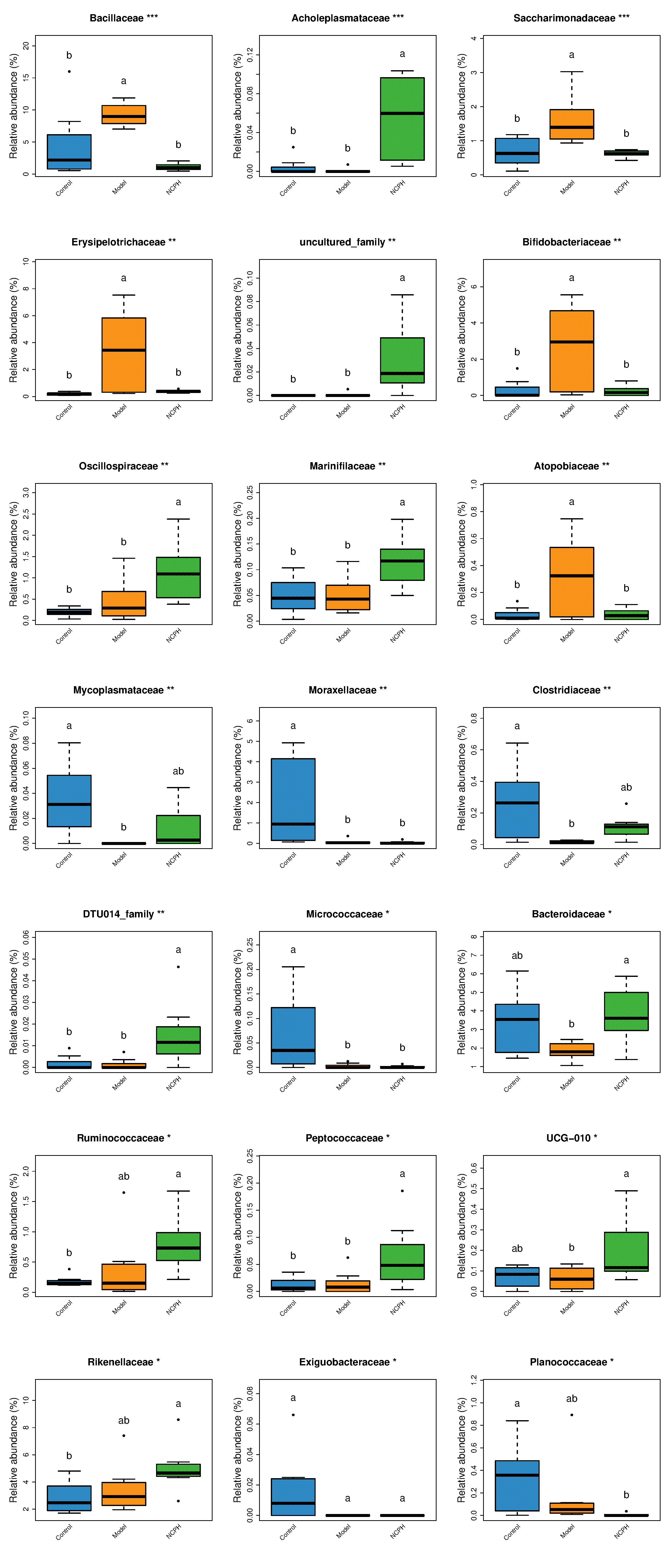


**Supplementary Figure 13** Box plot of community structure analysis at the family level.


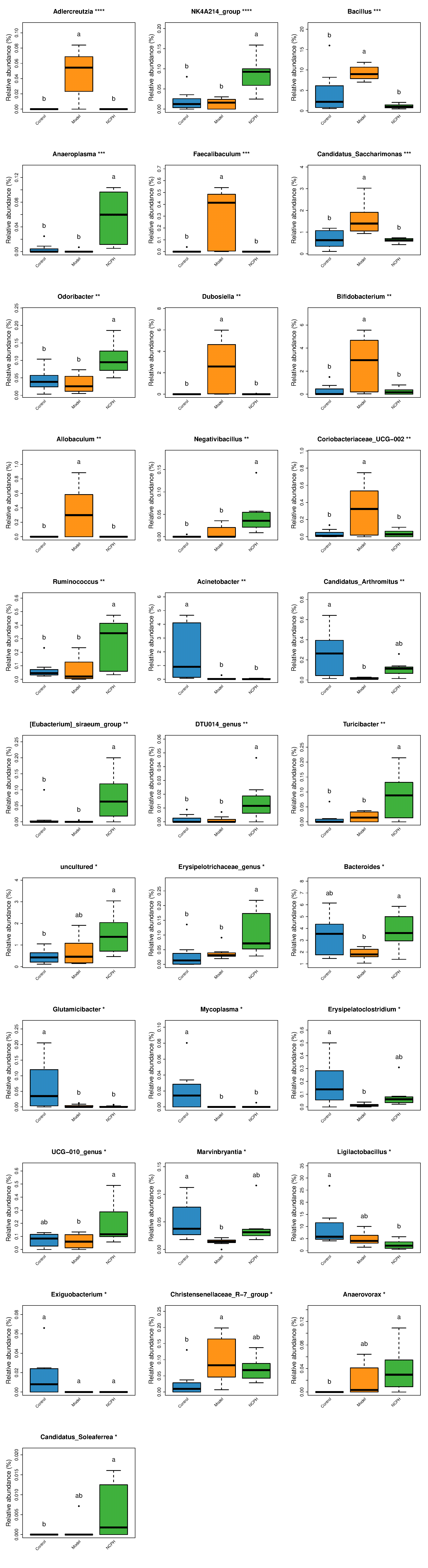


**
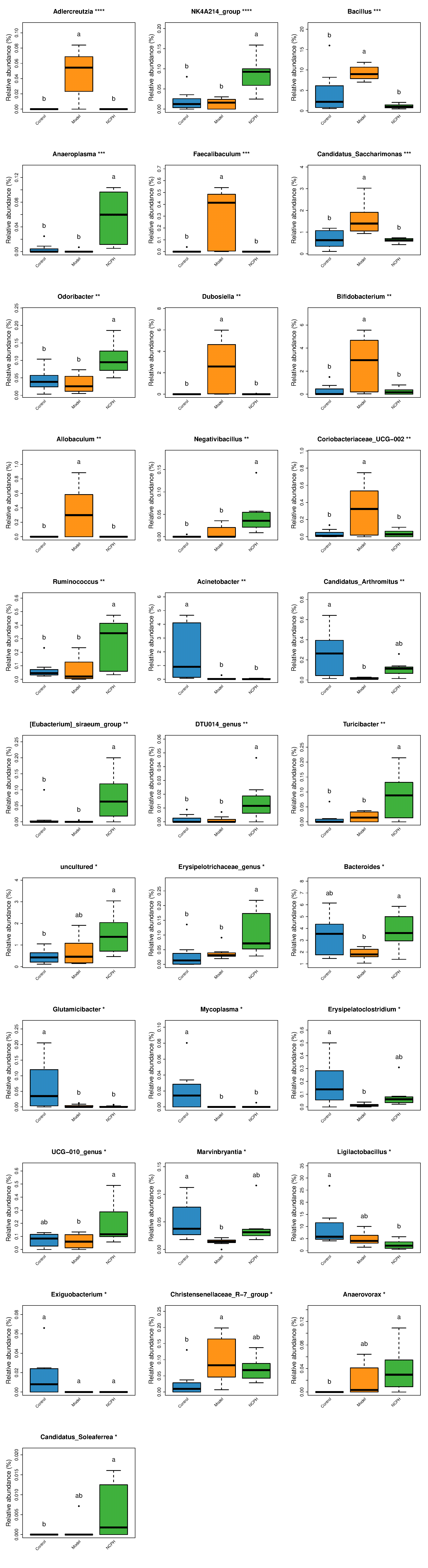
**

**Supplementary Figure 14** Box plot of community structure analysis at the genus level.


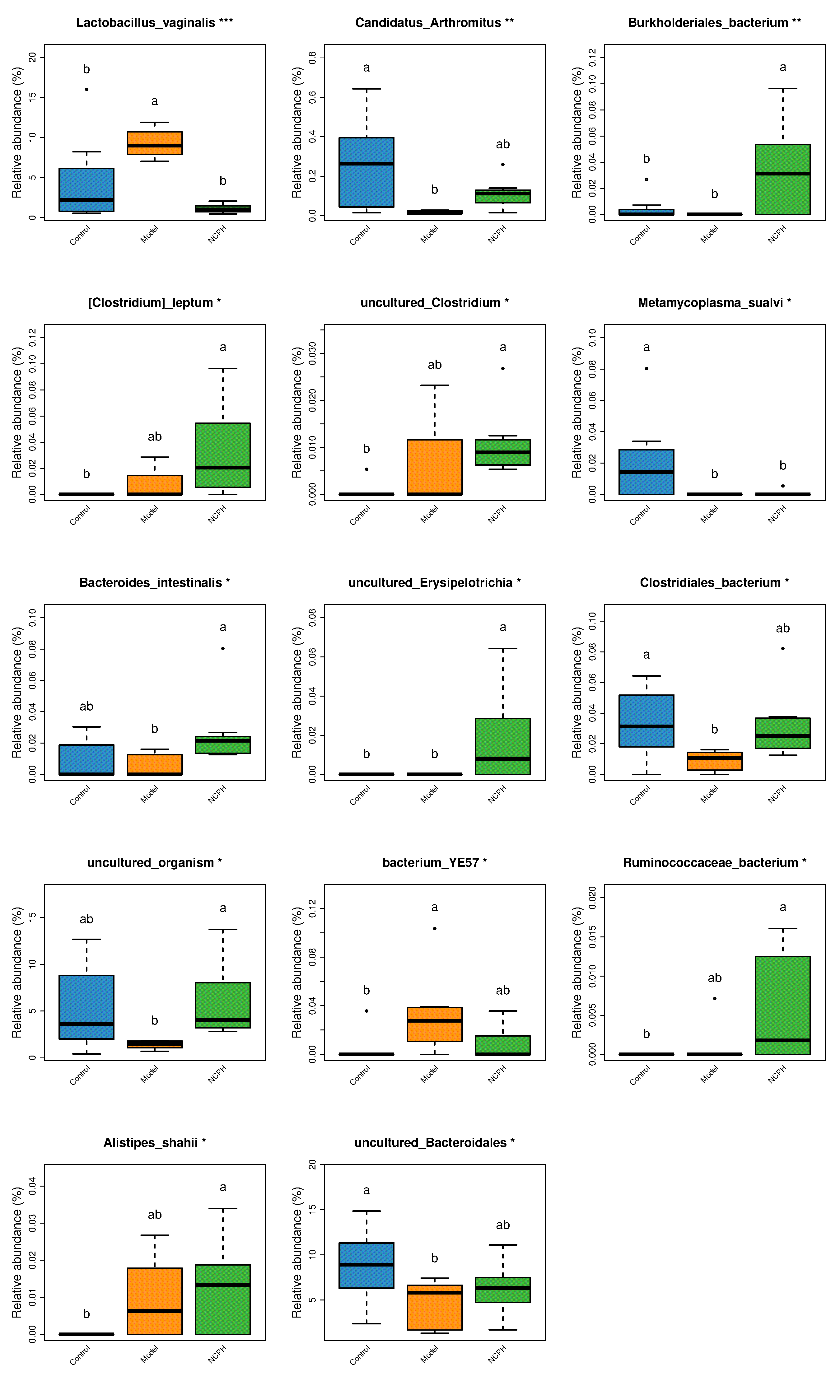


**Supplementary Figure 15** Box plot of community structure analysis at the species level.

**Supplementary Table. 1** Identified differential metabolites in positive ion mode

| No. | RT/min | Metabolites | Molecular formula | Molecular weight | VIP | P | Trend |
| --- | --- | --- | --- | --- | --- | --- | --- |
| 1 | 1.29 | Glycerophosphocholine | C_8_H_20_NO_6_P | 258.1104 | 1.46 | 0.00 | ↑ |
| 2 | 1.39 | Beta-Guanidinopropionic acid | C_4_H_9_N_3_O_2_ | 132.0770 | 1.13 | 0.04 | ↓ |
| 3 | 1.59 | Pyrrolidine | C_4_H_9_N | 72.0806 | 1.81 | 0.04 | ↓ |
| 4 | 10.34 | LysoPC(18:1(11Z)/0:0) | C_26_H_52_NO_7_P | 544.3406 | 1.42 | 0.02 | ↓ |
| 5 | 12.71 | (Halogen)-(Z)-2-(5-Tetradecenyl)cyclobutanone | C_18_H_32_O | 282.2789 | 1.60 | 0.02 | ↓ |
| 6 | 12.85 | 13-HODE | C_18_H_32_O_3_ | 279.2320 | 1.07 | 0.01 | ↓ |
| 7 | 13.54 | LysoPC(17:0/0:0) | C_25_H_52_NO_7_P | 510.3559 | 1.26 | 0.00 | ↑ |
| 8 | 13.85 | 17-HDoHE | C_22_H_32_O_3_ | 327.2320 | 1.81 | 0.00 | ↓ |
| 9 | 14.15 | (1xi,4xi,6xi)-Carvone oxide | C_10_H_14_O_2_ | 131.0853 | 1.39 | 0.03 | ↓ |
| 10 | 14.15 | Retinyl ester | C_20_H_30_O_2_ | 303.2324 | 1.48 | 0.01 | ↓ |
| 11 | 14.15 | Annoglabasin E | C_20_H_32_O_3_ | 343.2245 | 1.53 | 0.00 | ↓ |
| 12 | 14.37 | LysoPC(22:4(7Z,10Z,13Z,16Z)/0:0) | C_30_H_54_NO_7_P | 536.3707 | 1.04 | 0.03 | ↑ |
| 13 | 14.77 | Palmitoleoylethanolamde | C_18_H_35_NO_2_ | 280.2635 | 1.09 | 0.04 | ↓ |
| 14 | 19.39 | Linolenelaidic acid | C_18_H_30_O_2_ | 279.2322 | 1.66 | 0.00 | ↑ |
| 15 | 19.41 | Calendic acid | C_18_H_30_O_2_ | 261.2211 | 1.55 | 0.04 | ↑ |
| 16 | 20.56 | 1,1,2-Trimethyl-3,5-bis(1-methylethenyl)cyclohexane | C_15_H_26_ | 239.2370 | 1.11 | 0.01 | ↑ |
| 17 | 21.33 | anhydroretinol | C_20_H_28_ | 269.2264 | 1.06 | 0.01 | ↑ |
| 18 | 21.90 | 5Z-Dodecenoic acid | C_12_H_22_O_2_ | 199.1690 | 1.72 | 0.00 | ↑ |
| 19 | 21.91 | 2-Methyl-3-(2-pentenyl)-2-cyclopenten-1-one | C_11_H_16_O | 197.1538 | 1.56 | 0.00 | ↑ |
| 20 | 21.91 | Ethyl (4Z)-4,7-octadienoate | C_10_H_16_O_2_ | 151.1117 | 1.61 | 0.00 | ↑ |
| 21 | 21.91 | Geijerone | C_12_H_18_O | 211.1693 | 1.57 | 0.00 | ↑ |
| 22 | 21.91 | (R)-Carvone | C_10_H_14_O | 183.1377 | 1.63 | 0.01 | ↑ |
| 23 | 21.91 | (E)-5,8-Megastigmadien-4-one | C_13_H_20_O | 225.1850 | 1.67 | 0.00 | ↑ |
| 24 | 21.91 | (Z)-alpha-Irone | C_14_H_22_O | 207.1744 | 1.79 | 0.00 | ↑ |
| 25 | 21.91 | Linoleic acid | C_18_H_32_O_2_ | 281.2482 | 1.59 | 0.01 | ↑ |
| 26 | 21.92 | 1-Cyclopropyl-4-methyl-1,3-cyclohexanediol | C_10_H_18_O_2_ | 171.1379 | 1.70 | 0.00 | ↑ |
| 27 | 23.36 | Dihomo-gamma-linolenic acid | C_20_H_34_O_2_ | 307.2635 | 2.07 | 0.05 | ↑ |
| 28 | 25.07 | Palmitic acid | C_16_H_32_O_2_ | 257.2476 | 1.80 | 0.00 | ↓ |
| 29 | 27.12 | 2-Hexylidenecyclopentanone | C_11_H_18_O | 199.1692 | 2.18 | 0.00 | ↑ |
| 30 | 27.13 | Linalool oxide III | C_10_H_18_O_2_ | 153.1271 | 2.11 | 0.00 | ↑ |
| 31 | 27.14 | (Z)-13-Octadecenoic acid | C_18_H_34_O_2_ | 283.2638 | 2.10 | 0.00 | ↑ |
| 32 | 27.16 | 2-Hydroxy-2,6,6-trimethylcyclohexanone | C_9_H_16_O_2_ | 139.1113 | 2.06 | 0.00 | ↑ |
| 33 | 27.39 | Propylene glycol stearate | C_21_H_42_O_3_ | 384.3474 | 2.01 | 0.01 | ↓ |
| 34 | 27.75 | erythro-6,8-Pentacosanediol | C_25_H_52_O_2_ | 429.3768 | 1.47 | 0.01 | ↓ |
| 35 | 3.43 | (R)-Amphetamine | C_9_H_13_N | 136.1117 | 1.37 | 0.02 | ↓ |
| 36 | 30.94 | Tricosanoylglycine | C_25_H_49_NO_3_ | 429.3967 | 1.60 | 0.00 | ↓ |
| 37 | 32.00 | LysoPC(0:0/18:1(9Z)) | C_26_H_52_NO_7_P | 504.3445 | 1.80 | 0.00 | ↓ |
| 38 | 7.51 | Eremopetasinorol | C_13_H_20_O_2_ | 250.1776 | 1.26 | 0.03 | ↑ |
| 39 | 8.05 | Polyoxyethylene 40 monostearate | C_20_H_40_O_3_ | 346.3317 | 1.17 | 0.01 | ↓ |

**Supplementary Table 2** Identified differential metabolites in negative ion mode

| No. | RT/min | Metabolites | Molecular formula | Molecular weight | VIP | P | Trend |
| --- | --- | --- | --- | --- | --- | --- | --- |
| 1 | 1.37 | Dihydro-5-propyl-2(3H)-furanone | C_7_H_12_O_2_ | 165.0361 | 1.07 | 0.02 | ↓ |
| 2 | 1.44 | N-Glycolylneuraminic acid | C_11_H_19_NO_10_ | 306.0788 | 1.17 | 0.00 | ↓ |
| 3 | 1.51 | 1,2-Dichloroethane | C_2_H_4_C_l2_ | 96.9605 | 1.02 | 0.00 | ↑ |
| 4 | 10.28 | 5-Oxooctadecanoic acid | C_18_H_34_O_3_ | 279.2336 | 1.12 | 0.00 | ↑ |
| 5 | 11.14 | LysoPE(20:1(11Z)/0:0) | C_25_H_50_NO_7_P | 542.3039 | 1.57 | 0.00 | ↑ |
| 6 | 11.77 | Phosphate | H_3_O_4_P | 78.9591 | 1.11 | 0.00 | ↑ |
| 7 | 11.87 | LysoPI(18:2(9Z,12Z)/0:0) | C_27_H_49_O_12_P | 595.2903 | 1.64 | 0.02 | ↑ |
| 8 | 12.91 | LysoPC(P-16:0/0:0) | C_24_H_50_NO_6_P | 514.3078 | 1.02 | 0.00 | ↑ |
| 9 | 12.96 | LysoPC(17:0/0:0) | C_25_H_52_NO_7_P | 544.3185 | 1.62 | 0.01 | ↑ |
| 10 | 14.64 | 1-(11Z,14Z-eicosadienoyl)-glycero-3-phosphate | C_23_H_43_O_7_P | 507.2737 | 1.30 | 0.01 | ↑ |
| 11 | 19.23 | 16-Hydroxyhexadecanoic acid | C_16_H_32_O_3_ | 271.2284 | 1.57 | 0.00 | ↑ |
| 12 | 29.85 | PC(20:3(5Z,8Z,11Z)/18:1(11Z)) | C_46_H_84_NO_8_P | 830.5922 | 1.38 | 0.03 | ↓ |
| 13 | 32.17 | PG(20:3(6,8,11)-OH(5)/20:2(11Z,14Z)) | C_46_H_81_O_11_P | 885.5522 | 1.78 | 0.05 | ↑ |
| 14 | 4.38 | Phenol sulphate | C_6_H_6_O_4_S | 172.9919 | 1.13 | 0.01 | ↑ |
| 15 | 5.89 | Ginsenoside F2 | C_42_H_72_O_13_ | 829.4608 | 1.66 | 0.00 | ↑ |
| 16 | 6.32 | Ginsenoside Rg3 | C_42_H_72_O_13_ | 829.4629 | 1.49 | 0.00 | ↑ |
| 17 | 6.36 | PG(22:5(4Z,7Z,10Z,13Z,19Z)-O(16,17)/i-18:0) | C_46_H_79_O_11_P | 819.4339 | 1.95 | 0.01 | ↓ |
